# Synthesis and structure/properties characterizations of four polyurethane model hard segments

**DOI:** 10.1098/rsos.180536

**Published:** 2018-07-25

**Authors:** Lei Jiang, Zhiyong Ren, Wei Zhao, Wentao Liu, Hao Liu, Chengshen Zhu

**Affiliations:** 1 School of Materials Science and Engineering, Zhengzhou University, Zhengzhou 450001, People's Republic of China; 2 High and New Technology Research Center of Henan Academy of Sciences, Zhengzhou 450002, People's Republic of China

**Keywords:** polyurethane hard segment, structural characterization, crystallization behaviour, hydrogen bonding

## Abstract

Four model polyurethane (PU) hard segments were synthesized by reaction of butanol with four typical diisocyanates. The four diisocyanates were aromatic 4,4′-diphenylmethane diisocyanate (4,4′-MDI) and MDI-50 (50% mixture of 2,4′-MDI and 4,4′-MDI), cycloaliphatic 4,4′-dicyclohexylmethane diisocyanate (HMDI) and linear aliphatic 1,6-hexamethylene diisocyanate (HDI). FTIR, ^1^H NMR, ^13^C NMR, MS, X-ray and DSC methods were employed to determine their structures and to analyse their crystallization behaviours and hydrogen bonding interactions. Each of the four PU compounds prepared in the present work displays unique spectral characteristics. The FTIR bands and NMR resonance peaks assigned in the four samples thus provide a reliable database and starting point for investigating the relationship between hard segment structure and the crystallization and hydrogen bonding behaviour in more complex-segmented PU compositions.

## 1. Introduction

Polyurethanes (PUs) are a class of segmented copolymers composed of soft and hard segments. The soft segment is usually a polyether or polyester polyol while the hard segment is composed of a diisocyanate and chain extender. The soft segment provides elasticity, whereas the hard segment contributes strength and rigidity through physical cross-linking points. In the past several decades, PUs have found various applications. These range [1,2] from foams, elastomers, adhesives, paints and fibres (spandex) and so on to special coatings [3–5]. For each application, the PU compositions selected afford needed physical characteristics that may be uniquely required in the particular application. Therefore, the research on PU has been a hot topic in polymer research [6–8]. Previous investigations have shown that the relationship between structure and properties in PUs is of course largely determined by selection of starting materials, but also by the polymerization method [9,10], degree of phase separation [11,12], morphology [13,14] and by extent of crystallization [15,16] and hydrogen bonding [17,18] that are present in the final polymer. While the major soft segment component structure has a great influence on properties [19,20], the minor hard segment component structure also affects properties. This is because chemically bound hard and soft segment structures act together [21–23] to affect degree of phase separation, crystallization, morphology and hydrogen bonding. As these factors are known to control physical properties, a deep understanding of hard segment interactions is of important significance to future control of general PU composition in order to obtain physical properties matching requirements for specific applications.

However, the effect of hard segment structure on physical properties is also closely related to preparation conditions and sample heat history. Therefore, preparation of ‘pure’ hard segment with uniform chain length (excluding soft segment) makes it possible to accurately quantify the behaviour and properties of pure hard segments and to compare different hard segment structures. This knowledge will provide a sound basis for predicting the properties of hard segments in more complex-segmented PU compositions.

There are some reports on the relationship between different hard segments (based on different diisocyanates) and properties in PU. Studies have included hard segments consisting of MDI and 1,4-butanediol (BDO) [24,25], HDI and BDO [26], HMDI and BDO [27], and MDI-50 and BDO [28]. The hard segments in these studies, however, were conducted on compositions also containing soft segment. Using monodisperse hard segments (without chain extenders) [29] is another way of investigating the effect of hard segment structure on properties of segmented PUs. We are now studying the pure hard segments only (here we consider them as model hard segments); for the present we thus exclude any influence of soft segment. Our previous research work has dealt with model polyurethaneurea compounds prepared by reacting HMDI with several amine chain extenders [30]. The model PUs synthesized by different diisocyanates reacting with a mono-hydroxy compound (here butanol (BO)) were studied in the present work, because BO actually acts as a terminator. This is important because model hard segments with known molecular weight and fixed chain length could be obtained. However, in order to fully validate the use of model compounds in predicting structural and spectral behaviour in actual segmented PU structures, we plan to carry out additional studies. In future research we will conduct a structured, detailed study of a series of PUs of increasing complexity, by means of synthetic and characterization work that will extend the results of this paper. The study here described thus represents the first in a planned series.

Among the diisocyanates normally used in PU preparations, 4,4′-diphenylmethane diisocyanate (4,4′-MDI) is the most commonly used aromatic diisocyanate [31]. This compound has been found useful in various PU products, such as elastomers, especially spandex. 4,4′-MDI is the main component among its three isomers, and therefore, is called pure MDI. The distance between the two NCO groups in 4,4′-MDI is relatively long and there are no intervening substituents, so the reactivity in the two NCO groups is similar [32]. The two phenyl rings in MDI endow PU elastomers and spandex yarns with excellent properties.

MDI-50 is the 50/50 mol% mixture of 4,4′-MDI and 2,4′-MDI, and is referred to as MDI-50 [28]. Since it is hard to obtain pure 2,4-MDI, MDI-50 is the commonly used mixture. Since the structure of 2,4′-MDI is asymmetric, its crystallinity is much weaker than 4,4-MDI. In addition, the reactivity of the NCO at the 2 position is lower than that at the 4 position in the phenyl ring; also, due to its liquid state at room temperature, MDI-50 is more easily controlled and handled. Therefore, MDI-50 can be used as a partial substitute for 4,4-MDI in some coating, adhesive and elastomer compositions.

4,4-dicyclohexylmethane diisocyanate (HMDI, also called H_12_MDI), the hydrogenated MDI, is another useful isocyanate for PU products owing to its excellent light stability and hydrolysis resistance. Like MDI, HMDI has three isomers, which have obvious effects on the properties of PU made from them [33]. Therefore, when describing the HMDI-based PU, the isomer content must be specified.

1,6-hexamethylene diisocyanate (HDI) was used in early work with PU. HDI is also light stable due to absence of aromatic rings in its structure, but its reactivity is lower than that of aromatic isocyanates. Since the structure of HDI consists of an aliphatic chain, the HDI-based PU can be very easily crystallized and can be used in some special applications [31].

In summary, in the present work, these four typical diisocyanates were selected for synthesizing PU model hard segments via the reaction with BO. Fourier transform infrared spectroscopy (FTIR), proton nuclear magnetic resonance (^1^H NMR) and carbon-13 nuclear magnetic resonance (^13^C NMR) as well as mass spectroscopy (MS) were used to determine their structures. Wide angle X-ray diffractometry (WAXD) and differential scanning calorimetry (DSC) were used for characterizing their crystallization behaviour. Spectral characteristics and crystallization behaviour of each of the four model hard segments were unique. Thus, the assignment of the bands in both FTIR and NMR can be used as a database of key characteristics. These data can also provide a foundation for further study of the relationships between structure and crystallization and hydrogen bonding (H-bonding) interactions in more complicated segmented PUs, as noted above.

## 2. Material and methods

### 2.1. Materials

**Figure 1.**
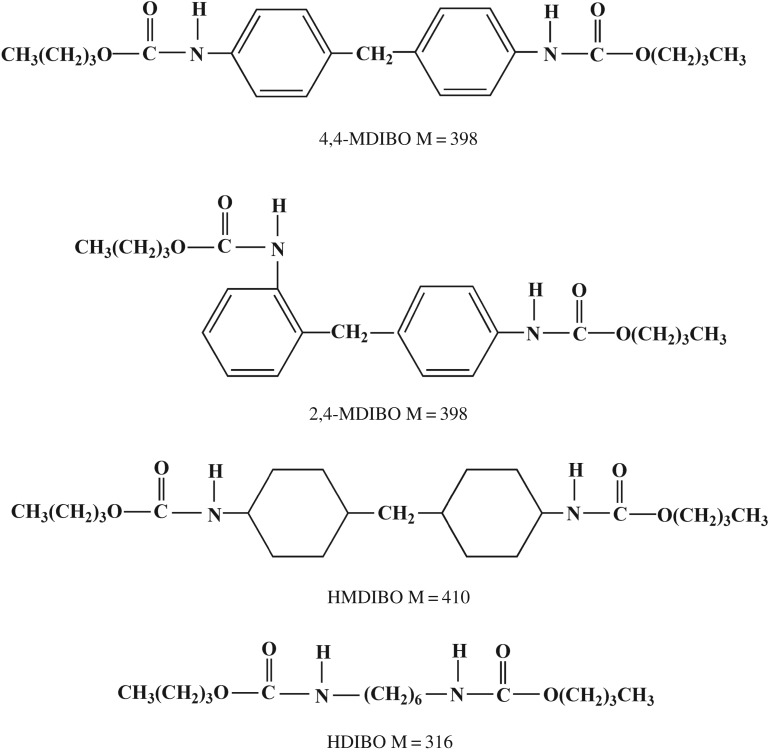
Structure scheme of four PU model hard segments.

4,4-MDI, MDI-50, HDI and HMDI were obtained from Wanhua Chemical Group Co. Ltd, China. BO was used as chain terminator and butanone was used as solvent during syntheses. Both were analytical grade reagents, which were dehydrated under 4A molecular sieves and distilled before being used. The structures of the four model hard segments are seen in figure 1.

### 2.2. Syntheses

Syntheses of the model hard segments were conducted by solution polymerization. Stoichiometric (mole ratio of BO over diisocyanate is 2.1 : 1) BO; each diisocyanate (4,4-MDI, MDI-50, HMDI and HDI) was added, in turn, into a three-neck, round bottom flask equipped with an overhead stirrer, reflux column and nitrogen inlet. The reaction was conducted at 70–80°C in butanone for about 2 h until NCO was completely reacted. Then the solvent was removed and the products thus synthesized were purified by recrystallization to further remove BO residue before being characterized by various instruments.

The four model PU hard segments synthesized by BO with four different diisocyanates were abbreviated as 4,4-MDI_BO, MDI-50_BO, HMDI_BO and HDI_BO, respectively.

### 2.3. Characterization

FTIR spectra were obtained using SHIMADZU FTIR-8700 spectrophotometer. The frequency range covered was from 4000 to 400 cm^−1^ by averaging 32 scans at a resolution of 4 cm^−1^.

^1^H and ^13^C NMR spectra were recorded on a Bruker Avance III-400 MHz superconducting NMR spectrometer (400.1 MHz for ^1^H and 100.6 MHz for ^13^C) using DMSO-*d*_6_ as solvent at a concentration of approximately 5% (w/v) for ^1^H NMR and approximately 20% (w/v) for ^13^C NMR. All spectra were recorded at room temperature (298 K). Chemical shifts (*δ*) were given in parts per million with tetramethylsilane (0.1%) as internal standard.

MS data were obtained on micrOTOF-Q II mass spectrometer from Bruker Daltonics (Bremen, Germany). Ionization was achieved using an electrospray ionization source in the positive-ion mode. The MS data of the molecular ions were processed using the software Data Analysis v. 4.0.

DSC measurements were performed with STA-449C DSC-TG thermal analyser. Samples were run at 10°C min^−1^ in nitrogen, using about 10 mg of sample per run. Each sample was run from room temperature to 20°C above its melting point (*T*_m_), holding for 2 min, cooling to room temperature and then heating again to 20°C over *T*_m_. Thus, three DSC scans were obtained with two heating curves and one cooling curve. One of the heating curves is from the as-prepared compound, while another is the one in which the heat history was removed. The temperature of both the *T*_m_ and crystallization temperature (*T*_c_) is obtained by taking its peak value. In the present paper, only the data during second heating run was used.

X-ray characterization was performed with Bruker D8 Focus WAXD using a Co K*α* source between the angles 2*θ* = 10° and 50° with a scan rate of 1° min^−1^. The samples tested are as-prepared. For crystallinity calculation, the whole area was calculated first and the crystal area based on crystal peak was then calculated. The crystallinity was finally calculated by dividing the crystal area over the whole area via the software package in the diffractometer.

## 3. Results and discussion

### 3.1. Infrared spectroscopy

FTIR spectroscopy has proven to be a valuable technique for the identification of PU structure and H-bonding interactions between soft and hard segments as well as between hard segments alone [34–38]. This technique was used in the present study to identify the model PU structure together with NMR and MS.

**Figure 2.**
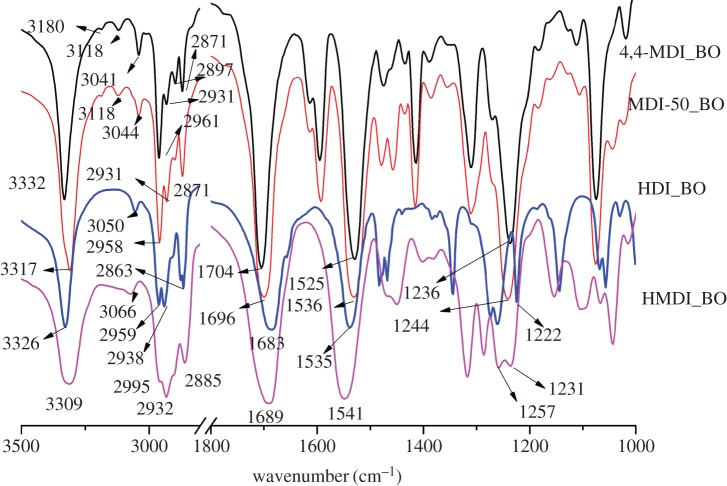
FTIR spectra of four model PU hard segments.

Figure 2 presents the FTIR spectra of four model PU hard segments including MDI_BO, MDI-50_BO, HMDI_BO and HDI_BO. It is seen that the four typical characteristic bands usually showing the formation of PU [34,35,38] are all obvious in the four samples, including the *v*N–H bands at 3309–3332 cm^−1^, the *v*C=O (amide I) band at 1683–1704 cm^−1^, the amide II band (*δ*N–H + *v*C–N) at 1529–1541 cm^−1^ and the amide III band (*v*C–N + *δ*N–H) at 1222–1257 cm^−1^. The frequency differences in different hard segments should result from their different structures, crystallizations and H-bonding interactions. The final band assignments for the four samples are shown in table 1.

**Table 1.** Main bands assignment of FTIR spectra in the four PU hard segments.

wavenumber (cm^−1^)	relative intensity	main assignment [34,35,38]
3332	vs	*v*N–H in 4,4-MDI_BO
3317	vs	*v*N–H in MDI-50_BO
3326	vs	*v*N–H in HDI_BO
3309	vs	*v*N–H in HMDI_BO
3180	vs	*v*C–H in 4,4-MDI_BO
3118	vs	*v*C–H in 4,4-MDI_BO
3041	vs	overtone of C=O in 4,4-MDI_BO
3118	vs	*v*C–H in MDI-50_BO
3044	vs	overtone of C=O in MDI-50_BO
3050	vs	overtone of C=O in HDI_BO
3066	vs	overtone of C=O in HMDI_BO
1704	vs	*v*C=O in MDI-50_BO
1696	vs	*δ*C=O in MDI-50_BO
1683	vs	*δ*C=O in HDI_BO
1689	vs	*δ*C=O in HMDI_BO
1525	vs	amide II in 4,4-MDI_BO
1536	vs	amide II in MDI-50_BO
1535	vs	amide II in HDI_BO
1541	vs	amide II in HMDI_BO
1236	vs	amide III in 4,4-MDI_BO
1244	vs	amide III in MDI-50_BO
1271	vs	amide III in HDI_BO
1231	vs	amide III in HMDI_BO
1257	vs	amide III in HMDI_BO

It can also be observed that there are two small bands between 3000 and 3300 cm^−1^ in the two aromatic-based PU samples while there is only one small band in the similar wavenumber range in two non-aromatic-based PUs. The band between 3041 and 3066 cm^−1^ can be attributed to the overtone of the urethane carbonyl [38]. The additional band at 3118 cm^−1^ can be attributed to *v*C=C–H of the phenyl ring in the two aromatic PU samples. We note that the overtone bands in the two aliphatic PU samples are higher than that in the two aromatic PU samples.

In addition, based on the ratio of *v*CH_2_ and *v*CH_3_, it is possible to judge the relative amount of CH_2_ and CH_3_. The four bands representing the CH_2_ and CH_3_ are almost the same for both 4,4-MDI_BO and MDI-50_BO (50% of 2,4-MDI), whereas the intensity of the bands at 2863 cm^−1^ in HDI_BO and the band at 2885 cm^−1^ in HMDI_BO is much stronger than that in the aromatic one, which is consistent with more CH_2_ in aliphatic and alicyclic compounds.

According to the wavenumbers, the N–H and C=O in the four model PUs are all in the H-bonded states. Usually, the wavenumber of *v*N–H and *v*C=O bands stands for the H-bonding strength: the lower the wavenumber is, the stronger the H-bonds would be. It can be seen from figure 2, however, that the changing trend of the wavenumber in *v*N–H and *v*C=O is not consistent. For MDI_BO, MDI-50_BO and HMDI_BO, the wavenumber of *v*N–H band is basically consistent with that of their *v*C=O band, namely, the higher the *v*N–H band is, the higher the *v*C=O would be. For HDI_BO, however, these two bands are not consistent. The *v*N–H is at 3326 cm^−1^, almost in the highest wavenumber among the four compounds, while the *v*C=O is at 1683 cm^−1^, which is in the lowest wavenumber.

Table 2 summarizes the bands associated with H-bonds including *v*N–H, *v*C–O as well as the amide II and amide III bands in the four model hard segments and also the sequence of both *v*N–H and *v*C=O.

**Table 2.** H-bonding related FTIR bands in four PU model hard segments. *v*C=O: HDI_BO < HMDI_BO < MDI-50_BO < 4,4-MDI_BO and *v*N–H: HMDI_BO < MDI-50_BO < HDI_BO < 4,4-MDI_BO.

	4,4-MDI_BO	MDI-50_BO	HDI_BO	HMDI_BO
*v*N–H	3332	3317	3326	3309
*v*C=O	1704	1696	1683	1689
amide II	1525	1536	1535	1541
amide III	1236	1244	1271	1257/1231

The amide II and III bands at about 1530–1540 cm^−1^ and 1220–1230 cm^−1^ are often used as additional proof of the formation of urethane structure and H-bond in PU [34,35,40]. These two bands are usually used only to confirm the urethane structure and the H-bond strength associated with *v*N–H and amide I band, namely, when *v*N–H is in lower wavenumber, both the amide II and III are in the higher wavenumber and vice versa. Figure 2 shows that three of the present four model PUs all correspond to this rule except HDI_BO, whose amide II is in the relatively higher wavenumber (1535 cm^−1^) while amide III is in the relatively lower wavenumber (1222 cm^−1^).

Since the preparation conditions and the samples' states (which would affect the wavenumbers of the bands) are the same in the present work, the above results show that the four characteristic wavenumbers of the four bands are associated with their different structures. It is also believed that these bands are also related to their different H-bonding and crystallization states, which will lay a basis for further study of the relationship between physical structure and H-bonding and crystallization in our planned future work.

### 3.2. Mass characterization

#### 3.2.1. 4,4-MDI_BO

The MS data (electronic supplementary material, figure S1) of 4,4-MDI_BO show that the molecular weights [M + H]^+^, [M + Na]^+^ and [M + K]^+^ of 4,4-MDI_BO are 399.2, 421.2 and 437.1, respectively. Its molecular formula is C_23_H_30_N_2_O_4_ with a corresponding molecular weight of 398 (figure 1).

#### 3.2.2. MDI-50_BO

MS data (electronic supplementary material, figure S2) of MDI-50_BO show that the molecular weights [M + H]^+^, [M + Na]^+^ and [M + K]^+^ of MDI-50_BO are 399.2, 421.2 and 437.1, respectively. Its molecular formula is C_23_H_30_N_2_O_4_ with a corresponding molecular weight of 398. Actually, 2,4-MDI in MDI-50 has the same molecular weight as 4,4-MDI (figure 1**)**.

#### 3.2.3. HDI_BO

MS data (electronic supplementary material, figure S3) of HDI_BO show that the molecular weights [M + H]^+^ and [M + Na]^+^ of 4,4-MDI_BO are 317.1 and 339.4, respectively. Its molecular formula is C_16_H_32_N_2_O_4_ with a corresponding molecular weight of 316 (figure 1).

#### 3.2.4. HMDI_BO

MS data (electronic supplementary material, figure S4) of HMDI_BO show that the molecular weights [M + H]^+^, [M + Na]^+^ and [M + K]^+^ of 4,4-MDI_BO are 411.2, 433.3 and 449.2, respectively. Its molecular formula is C23H_32_N_2_O_4_ with a corresponding molecular weight of 410 (figure 1).

### 3.3. NMR analyses

Electronic supplementary material, figures S5–S12 are the ^13^C NMR and ^1^H NMR spectra in the four PU model hard segments. The chemical shifts and the assignments for both ^1^H NMR and ^13^C NMR spectra are shown in tables 3–6. Together with MS data, it can be concluded that the four PU model hard segments are just the expected structure with high purity.

**Table 3.** ^1^H, ^13^C NMR data for 4,4-MDI_BO.

no.	*δ*_H_	*δ*_C_
1	0.92 t	14.04
2	1.37 m	19.08
3	1.6 m	31.08
4	4.06 t	64.21
5		154.12
6	9.50s	
7		137.62
8	7.37d	118.79
9	7.09d	135.85
10		129.26
11	3.79s	40.28

**Table 4.** ^1^H, ^13^C NMR data for MDI-50_BO.

no.	*δ*_H_	*δ*_C_
1, 23	0.88 ∼ 0.93 (dt)	14.04 (overlapped)
2, 22	1.31 ∼ 1.42 (dm)	19.03, 19.07
3, 21	1.53 ∼ 1.63 (dm)	31.08, 31.18
4, 20	4.0 ∼ 4.08 (dt)	64.20, 64.26
6, 7, 8, 9, 10, 11, 13, 14, 15, 16, 17, 18	7.03 ∼ 7.38 (phenyl H)	118.59, 118.79, 125.52, 126.07, 126.88, 129.27, 129.33, 130.35, 134.43, 135.85, 136.51, 137.62 (phenyl C)
12	3.79, 3.91	36.38
24, 25	8.83, 9.50 (ds)	
5, 19		154.10, 155.06

**Table 5.** ^1^H, ^13^C NMR data for HDI_BO.

no.	*δ*_H_	*δ*_C_
1	0.88 t	14.06
2	1.29 ∼ 1.36 m	19.07
8		29.85
3	1.51	31.27
4	3.92 t	63.64
5		156.80
6	7.02 t	
7	2.94 m	39.99
9	1.24 m	26.39

**Table 6.** ^1^H, ^13^C NMR data for HMDI_BO.

no.	*δ*_H_	^1^H–^1^HCOSY	*δ*_C_
1,1′	0.88 ∼ 0.90		14.02
2,2′	1.29 ∼ 1.36 m		19.09
3,3′, 4,4′			
8,8′, 9,9′			
10,10′, 11	1.05 ∼ 1.75		28.00, 28.07, 29.37, 31.29, 32.04, 32.26, 32.31, 33.02, 33.81
5,5′			156.01, 156.15
6,6′	6.93	3.48, 3.17	
7,7′	3.48, 3.17	6.93	50.27, 47.57

The detailed analyses of NMR spectra on the four PU model hard segments is available in the electronic supplementary material. Here only the brief ^13^C NMR analysis is presented, based on the relationship between chemical shift and structures [39,41].

#### 3.3.1. 4,4-MDI_BO

According to its structure (figure 1), 4,4-MDI_BO has 23 carbon atoms. Owing to its symmetric structure, there are 11 carbon atoms in each side except the methylene group in the centre. Since there are two identical groups of carbon atoms in the phenyl rings, the actual ‘independent’ carbon number is 10. Therefore, 10 different carbon chemical shifts are present. It can be seen from electronic supplementary material, figure S5 that there are exactly 10 peaks in its ^13^C NMR spectrum, and as the partial enlarged NMR figure shows, one of them at *δ*C40.28 is mixed in the chemical shift of DMSO-*d*_6_.

#### 3.3.2. MDI-50_BO

Since there is 50% of 2,4-MDI in MDI-50, the asymmetric structure in it will lead to more carbon peaks in its ^13^C NMR spectrum (electronic supplementary material, figure S7), due to not only the two different phenyl rings (4,4-MDI and 2,4-MDI), but also the aliphatic chains attached to it. Therefore, there are 23 different ‘independent’ carbon atoms in MDI-50 altogether. In addition, because there is still 50% of 4,4-MDI in MDI-50, the height of ^13^C peaks is not consistent with the pure 2,4-MDI.

#### 3.3.3. HDI_BO

Same as 4,4-MDI_BO, HDI_BO has also symmetric structure containing 16 carbon atoms. Therefore, there are eight ^13^C NMR peaks (electronic supplementary material, figure S9), of which one at *δ*C39.99 is hidden in the DMSO-*d*_6_ peaks.

#### 3.3.4. HMDI_BO

In the HMDI-BO structure (of whatever isomer content), there are 2 six-membered rings like cyclohexane. Therefore, the seemingly symmetrical atoms are actually asymmetrical in the spatial structure. That is, the reason why there are more chemical shifts than are observed in the case of a structure with only a single isomer; this can be verified from ^13^C NMR and ^1^H NMR spectra (electronic supplementary material, figures S11 and S12).

### 3.4. X-ray analysis

Electronic supplementary material, figures 13S–16S are the X-ray results for the four model hard segments. It is seen that all of the four types of hard segment compounds are highly crystallized with their own characteristic X-ray peaks. These results are due to their different relatively regular structures. It can also be observed from the ordinate values of X-ray figures that among the four samples, HDI_BO has the strongest intensity, whose ordinate value is over 140 000 while HMDI_BO has the relatively weaker intensity with the intensity of a bit more than 11 000 (ordinate value). Compared with 4,4-MDI_BO, MDI-50 containing 2,4-MDI_BO has not only the different crystal form (its crystalline peaks are somewhat different from 4,4-MDI_BO), but also much weaker intensity, as the highest intensity of 4,4-MDI_BO is at about 22 000 while MDI-50_BO is at only close to 13 000.

According to the crystallinity calculated based on the X-ray figure (electronic supplementary material, figures 13S–16S), the sequence of crystallinity in the four model hard segments (as-prepared) is seen in table 7.

**Table 7.** Crystallinity calculated based on X-ray results.

	HDI_BO	HMDI_BO	4,4-MDI_BO	MDI-50_BO
crystallinity %	100	77	72	65

The crystalline behaviour among the four samples are consistent with their structure characteristics, which have been also shown in the following DSC result.

### 3.5. DSC analysis

**Figure 3.**
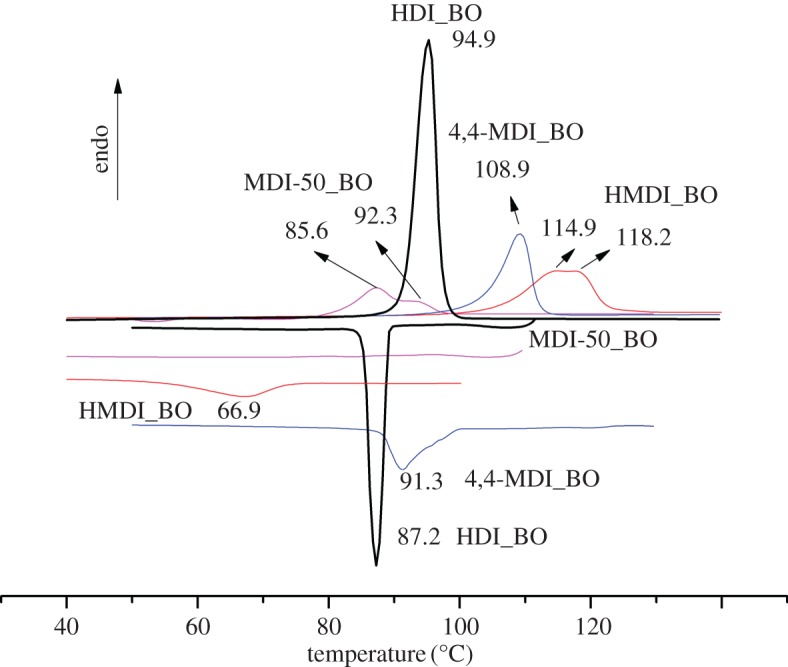
DSC scans of four model PU hard segments.

DSC scans of the four samples in the second heating run (after the thermal history was removed) are shown in figure 3. It can be seen that the *T*_m_ of the four samples are all clear, showing all of the four model PU compounds are crystallizable; however, the crystallization temperature and the shape of crystalline exothermic peaks as well as their supercooling degrees are various, showing their crystallization behaviours are quite different. Detailed analyses are as follows.

According to figure 3, HMDI_BO has the highest *T*_m_ while MDI-50_BO has the lowest; the *T*_m_ of 4,4-MDI_BO and HDI_BO are in the midst. Figure 3 also shows that three of the four samples have obvious *T*_c_, showing they have strong crystallization ability. Comparison of the values between *T*_m_ and *T*_c_ shows that they are different but have the same rule among three types of hard segments including HDI_BO, 4,4-MDI_BO and HMDI_BO, namely the lower the *T*_m_ is, the higher *T*_c_ would be and vice versa. It is quite reasonable for HDI_BO to have the highest *T*_c_, as the aliphatic chain has the stronger chain mobility than the aromatic chain, while lower *T*_m_ is also due to its aliphatic chain, as the general molecular interaction is weaker than the aromatic one.

As for MDI-50_BO, it seems that there is no indication of crystallization peak in the cooling process, suggesting it has poor crystallization ability during the cooling process in the present condition (10°C min^−1^ of cooling rate). This is not surprising because the branched NCO group (second position in the phenyl ring) in 2,4-MDI component in the isocyanate MDI-50 disturbs the chain regularity. However, because there is indeed a *T*_m_ peak (however small it is) in the second heat running, the sample must have crystallized during the crystallization process in the present work, possibly only the crystallization exothermic process is too long to be expressed in the DSC scan. The longer and weaker crystallization process makes the crystallization peak be hidden in the baseline. In this way, the crystallization curve is not sensitive to be recorded.

## 4. Conclusion

(1) The four PU model hard segments prepared in this work based on different representative diisocyanates (aromatic, cycloaliphatic and aliphatic structures) have clear structure with high purity.
(2) The detailed assignments in both FTIR bands and NMR resonance peaks can be taken as a database for studying more complicated segmented PUs.
(3) The crystallization behaviour in the four different types of PU hard segments is closely related to their different chemical structures. For HDI_BO, its crystallinity is almost complete, functioning as a small molecule with closest *T*_m_ and *T*_c_; for MDI-50_BO, 2,4-MDI content causes too large an influence on the crystallization of 4,4-MDI_BO, causing the crystallization process to be too long to be seen under the cooling rate of 10°C min^−1^.
(4) The relationship of *v*N–H and *v*C=O in 4,4-MDI_BO, MDI-50_BO and HMDI_BO have the same characteristic: lower *v*N–H occurs together with lower *v*C=O while these two bands, which are closely related to extent of H-bonding have a different relationship in the HDI_BO spectrum. This result may be associated with its crystallization behaviour. Further work on this point is planned for the future.

## Supplementary Material

Supplementary material

## Supplementary Material

Figure S1.tif

## Supplementary Material

Figure S2.tif

## Supplementary Material

Figure S3.tif

## Supplementary Material

Figure S4.tif

## Supplementary Material

Figure S5.jpeg

## Supplementary Material

Figure S6.jpeg

## Supplementary Material

Figure S7.jpeg

## Supplementary Material

Figure S7.jpeg

## Supplementary Material

Figure S8.jpeg

## Supplementary Material

Figure S9.jpeg

## Supplementary Material

Figure S10.jpeg

## Supplementary Material

Figure S11.jpeg

## Supplementary Material

Figure S13.gif

## Supplementary Material

Figure S14.gif

## Supplementary Material

Figure S15.gif

## Supplementary Material

Figure S16.gif

## Supplementary Material

Raw_data.rar

## Data accessibility

Data supporting this article are provided as the electronic supplementary material.
