# Supplementary material for: Synthesis and structure/properties characterizations of four polyurethane model hard segments
Source: R Soc Open Sci. 2018 Jul 25;5(7):180536. doi: 10.1098/rsos.180536 (PMC6083698; doi:10.1098/rsos.180536)
Supplement: Supplementary material [file rsos180536supp1.doc]

**Supplementary material**

Lei Jiang^1,2^, Zhiyong Ren^2^*, Wei Zhao^2^, Wentao Liu^1^, Hao Liu^1^, Chenshen Zhu^1^*

^1^School of Materials Science and Engineering, Zhengzhou University, Zhengzhou, China

^2^High & New Technology Research Center of Henan Academy of Sciences, Zhengzhou, China

*Correspondent authors: Email: *zyren23@yahoo.com, zhucs@zzu.edu.cn

**1. HRMS analysis**


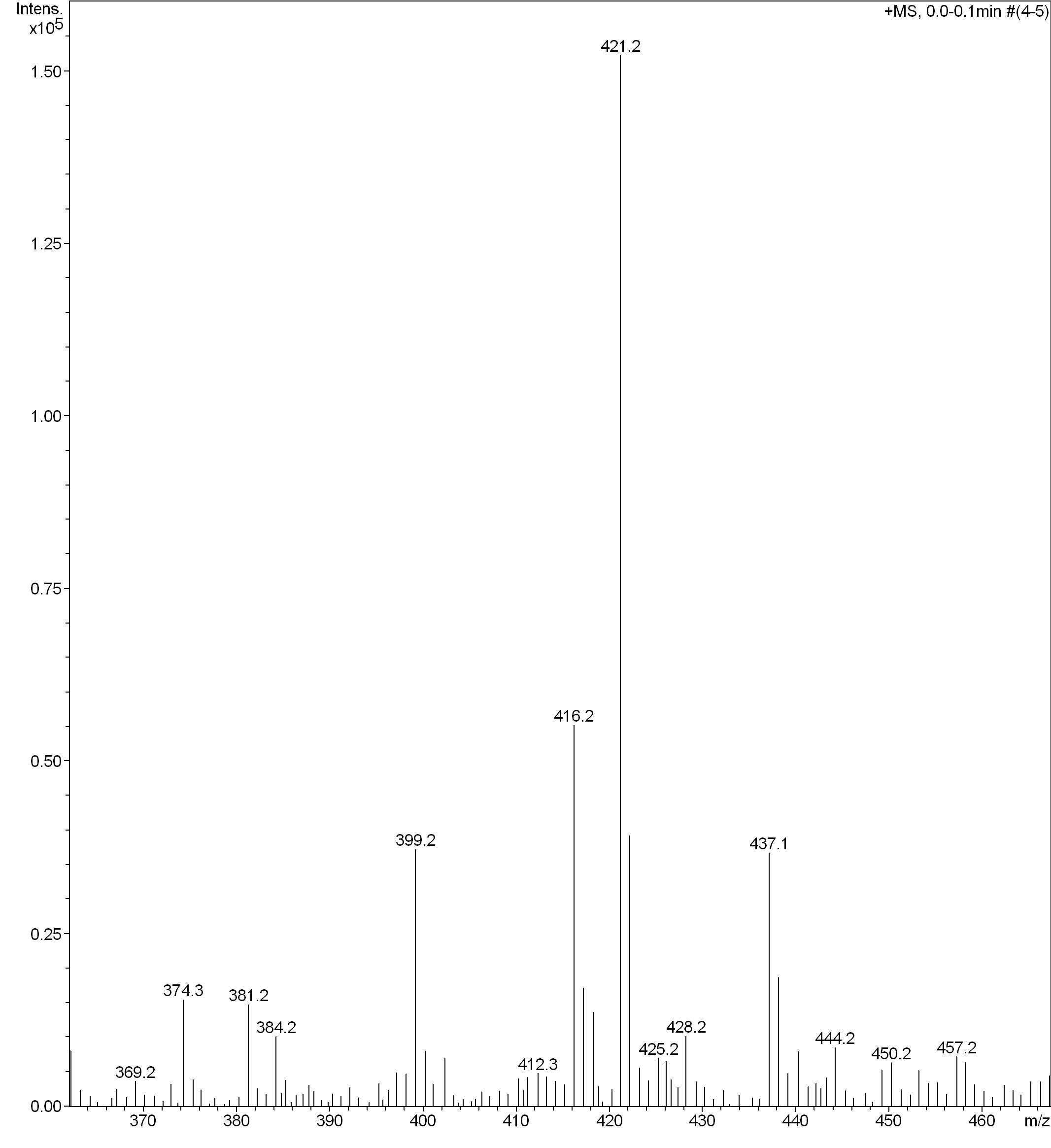


**Figure S1. HRMS spectra of 4,4-MDI_BO**

In Figure S1, the molecular weight of [M + H]+, [M + Na]+ and [M + K]+ of 4,4-MDI_BO are 399, 421 and 437, respectively.


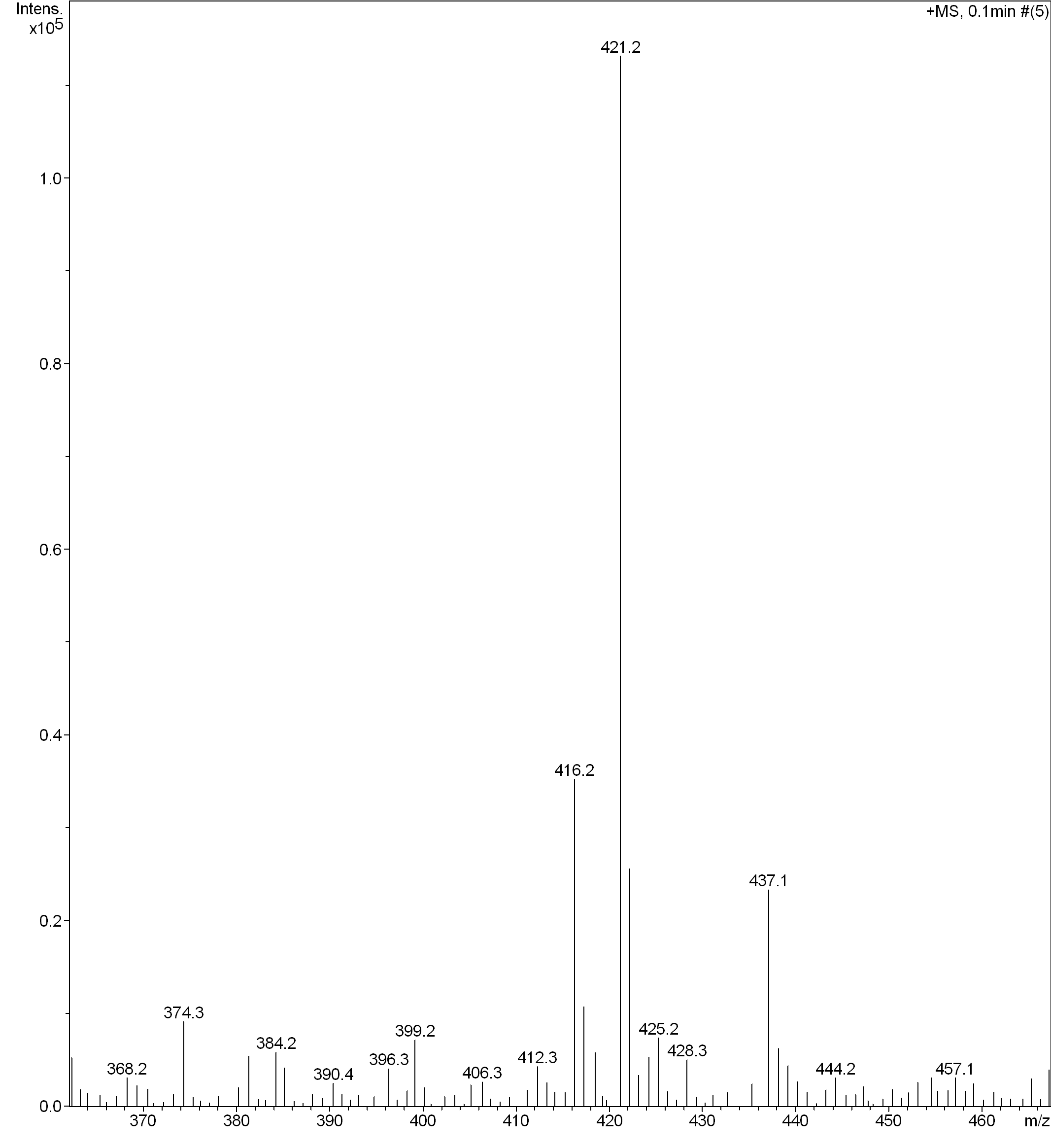


**Figure S2. HRMS spectra of MDI-50_BO**

In Figure S2, the molecular weight of [M + H]+, [M + Na]+ and [M + K]+ of MDI-50_BO are 399, 421 and 437, respectively, in which the molecular weight of 2,4-MDI_BO is the same as 4,4-MDI_BO, since they do have the same molecular weight, only 2,4-MDI in MDI-50 is another isomer of 4,4-MDI.


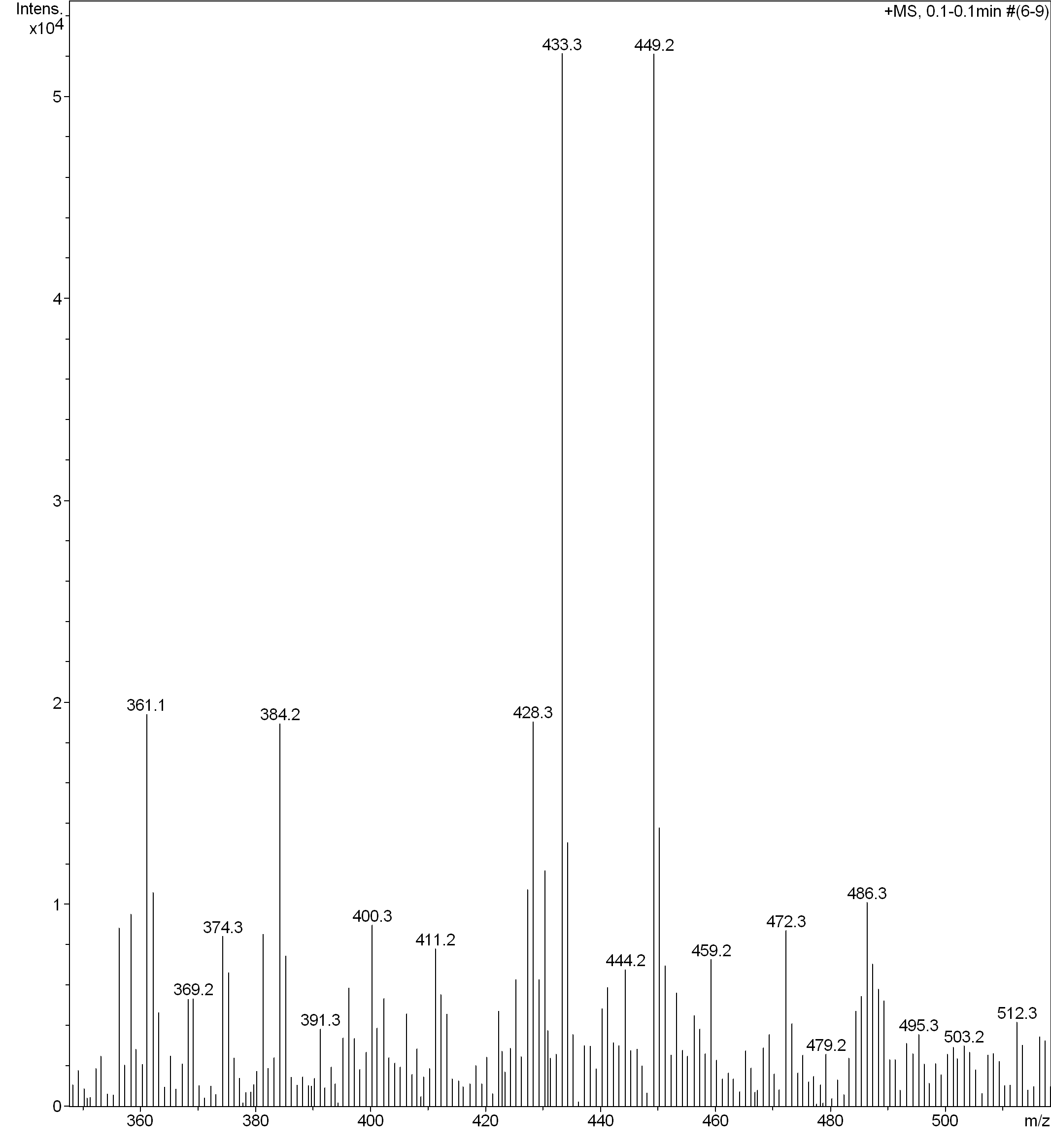


**Figure S3. HRMS spectra of HMDI_BO**

In Figure S3, the molecular weight of [M + H]+, [M + Na]+ and [M + K]+ of HMDI_BO are 411, 433 and 449, respectively.


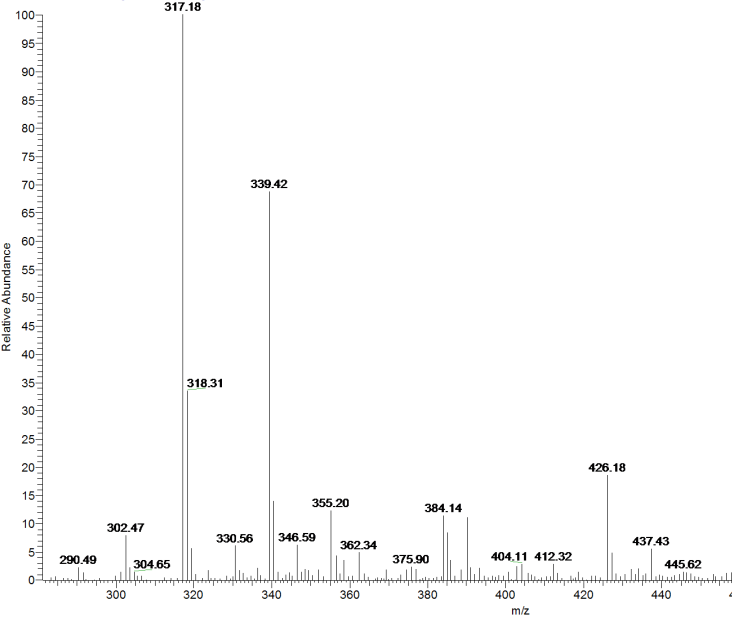


**Figure S4. HRMS spectra of HDI_BO**

In Figure S4, the molecular weight of [M + H]+ and [M + Na]+ of HDI_BO are 317 and 339, respectively.

**2. NMR analysis**

**1) ^13^C and ^1^H NMR spectra of 4,4-MDI_BO**

**
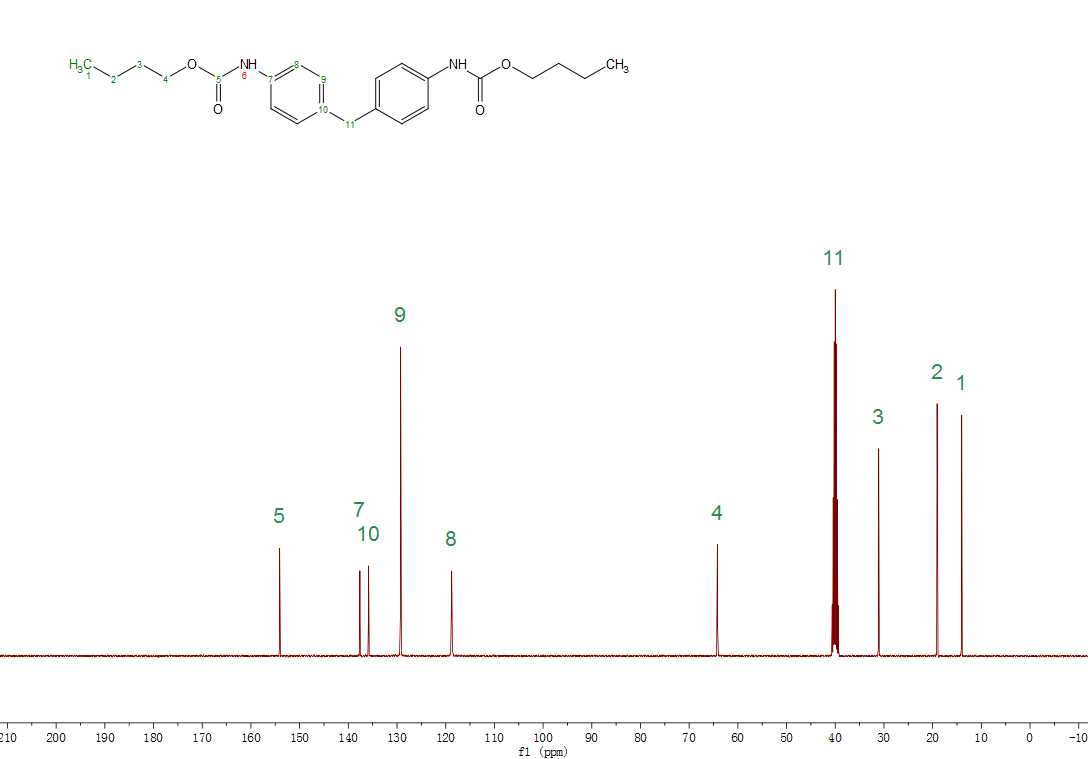
**

**Figure S5. ^13^C NMR spectrum of 4,4-MDI_BO**

**
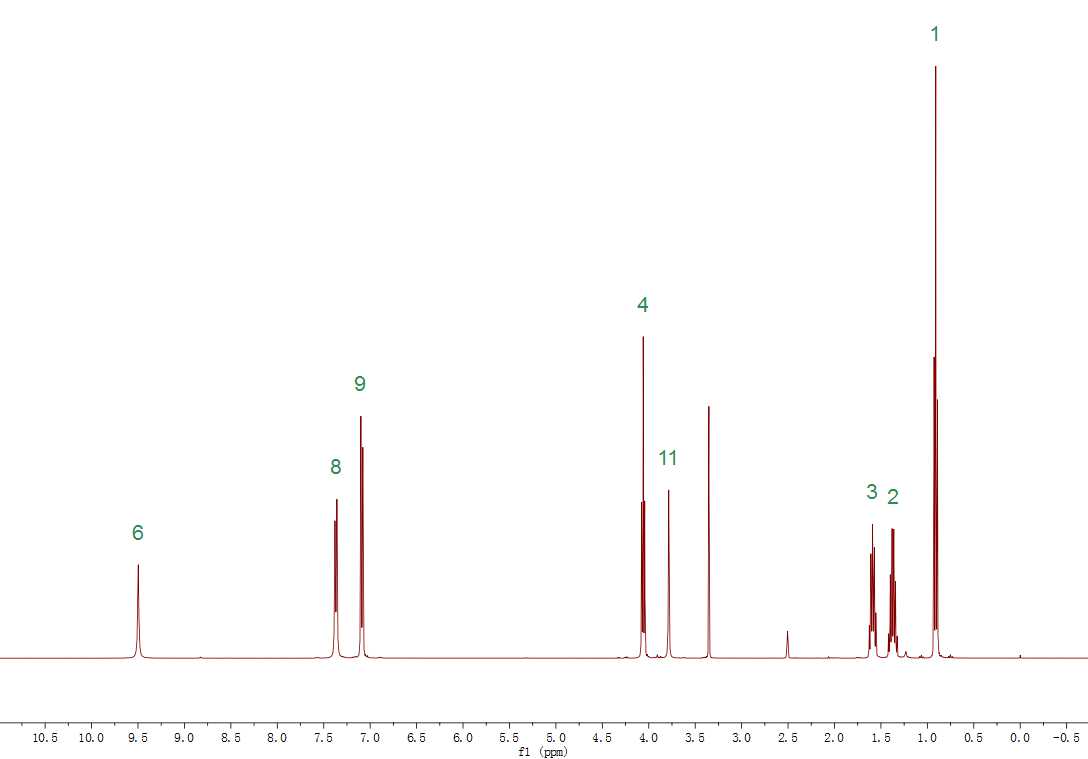
**

**Figure S6. ^1^HNMR spectrum of 4,4-MDI_BO**

According to the structure (Figure 1) of the synthesized 4,4-MDIBO, the product has symmetrical structure, so there should be 8 groups of hydrogen peaks and 10 bars of carbon peaks in ^1^H and ^13^C NMR spectra, respectively.

In the ^13^C NMR spectrum (Figure S5), the peak at δ_C_14.04 was attributes to the methyl carbon(C-1). The two peaks at δ_C_19.08 and δ_C_31.08 were assigned to two methylene carbons (C-2 and C-3), respectively. The peak at δ_C_19.08 belongs to C-2 and the peak at δ_C_31.08 belongs to C-3. The peak at δ_C_40.28 belongs to the methylene carbon (C-11) which is between the benzene rings. The peak at δ_C_64.21 belongs to the methylene carbon (C-4) which  connects to oxygen. The peaks at δ_C_129.26, δ_C_135.85, δ_C_118.79 and δ_C_137.62 belong to the four carbons on the benzene ring (C-7, C-8, C-9 and C-10), whose chemical shifts can be confirmed by the calculation of theoretical formula. And the peak at δ_C_154.12 belongs to the carbonyl carbon(C-5).

In the ^1^H NMR spectrum (Figure S6), the triplet peaks at δ_H_0.92 were attributed to methyl (H-1). The two multiple peaks at δ_H_1.37 and δ_H_1.6 were assigned to two methylenes (H-2 and H-3), respectively. The peak at δ_H_1.37 belongs to H-2 and the peak at δ_H_1.6 belongs to H-3. The single peak at δ_H_3.79 belongs to the methylene (H-11) which is between the two benzene rings. The triplet peaks at δ_H_4.06 belong to the methylene (H-4) which connects to oxygen. The two doublets at δ_H_7.09 and δ_H_7.37 belong to the two hydrogen atoms in the benzene ring (H-8 and H-9), respectively, whose chemical shifts can be calculated by theoretical formula. According to calculated result, H-9 is located in the high magnetic field and H-8 is located in the low magnetic field. The single peak at δ_H_9.50 belongs to the hydrogen attached to nitrogen atom. All NMR analytical data of 4,4-MDIBO are listed in Table 3 in the formal text.

**2) ^13^C and ^1^H NMR spectra of MDI-50_BO**

**
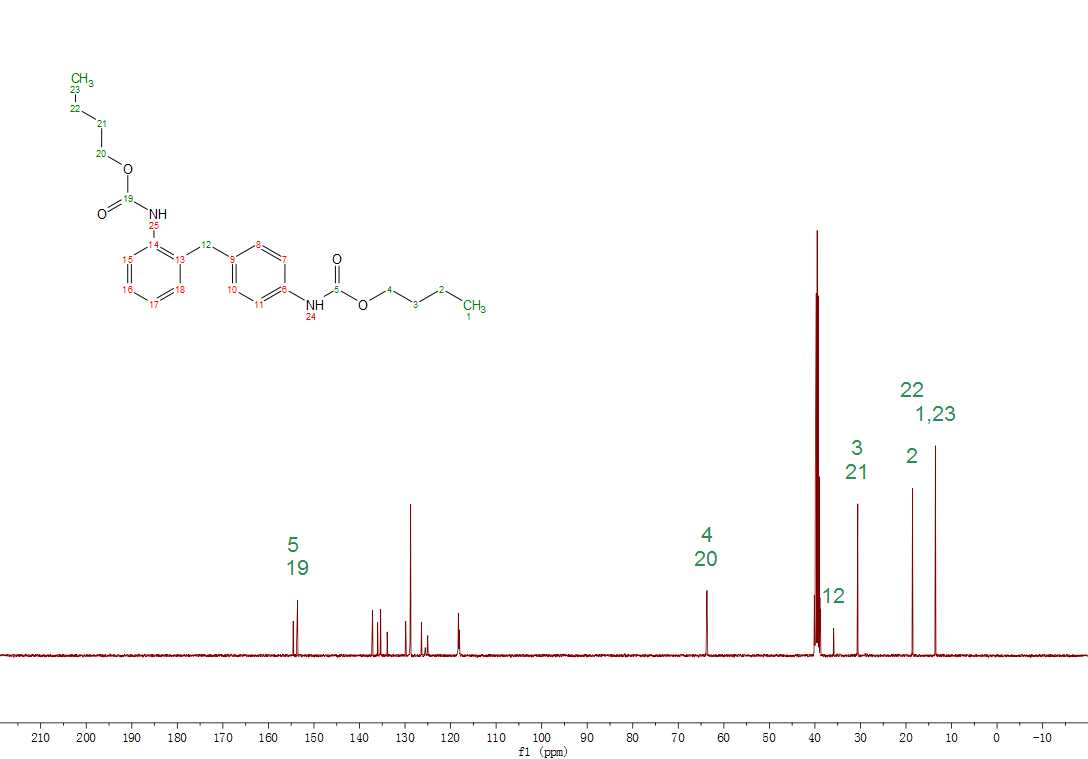
**

**Figure S7. ^13^C NMR spectrum of MDI-50_BO**

**
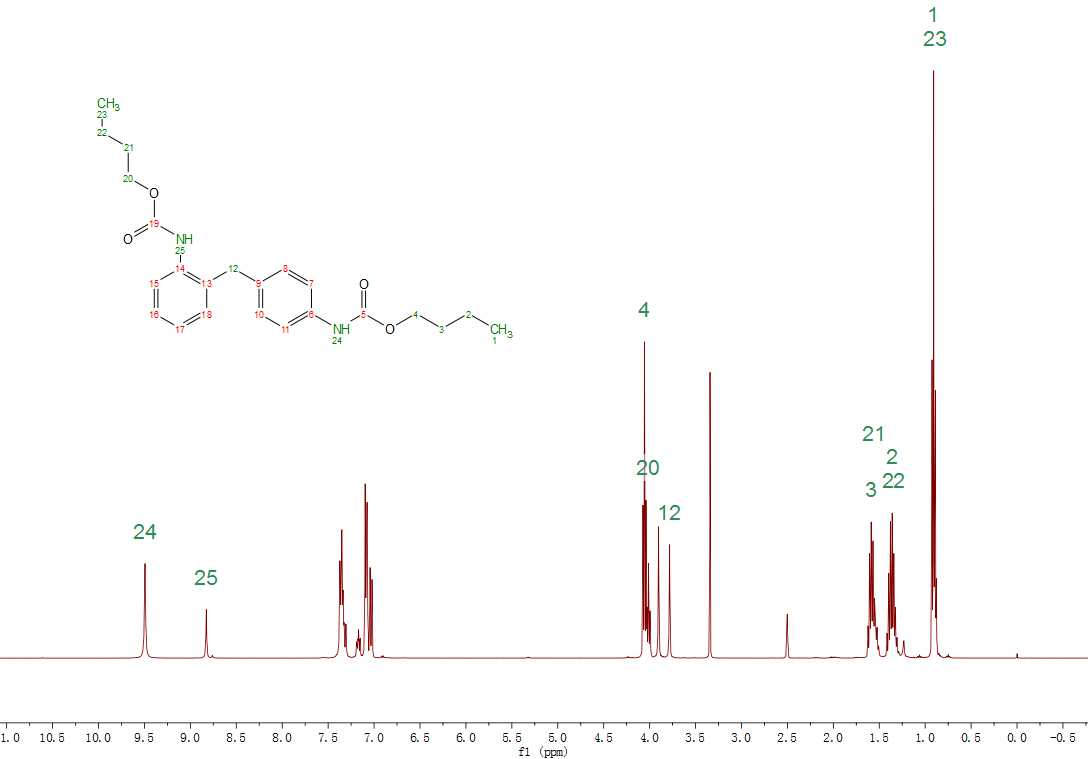
**

**Figure S8. ^1^H NMR spectrum of MDI-50_BO**

Compared with the structure of the 4,4-MDIBO (Figure 1), the structure of the 2,4-MDI_BO in MDI-50 is asymmetric. Although its ^1^H and ^13^C NMR spectra are a bit complex, it is also easy to be identified.

In the ^13^C NMR spectrum (Figure S7), the peaks at δ_C_14.04 attributes to two methyl carbons (C-1 and C-23) whose absorption peaks overlap. The peaks at δ_C_19.03 and δ_C_19.07 were assigned to two methylene carbons (C-2 and C-22), respectively. The peaks at δ_C_31.08 and δ_C_31.18 were assigned to two methylene carbons (C-3 and C-21). The peak at δ_C_36.38 belongs to the methylene carbon(C-12) which is between two benzene rings. The peaks at δ_C_64.20 and δ_C_64.26 belong to two methylene carbons (C-4 and C-20) which connects to oxygen. The peaks at δ_C_118.59, δ_C_118.79, δ_C_125.52, δ_C_126.07, δ_C_126.88, δ_C_129.27, δ_C_129.33, δ_C_130.35, δ_C_134.43, δ_C_135.85, δ_C_136.51 and δ_C_137.62 belong to twelve carbons on two benzene rings (C-7, C-8, C-10, C-11, C-15, C-16, C-17 and C-18). δ_C_154.1 and the peaks at δ_C_155.06 belong to two carbonyl carbon (C-5 and C-19).

In the ^1^H NMR spectrum (Figure S8), the two overlapping triplet peaks in the range of δ_H_0.88～0.93 were attributed to two methyls (H-1and H-23). The two overlapping multiple peaks in the range of δ_H_1.31～1.42 were assigned to two methylenes (H-2 and H-22). The two overlapping multiple peaks in the range of δ_H_1.53～1.63 were assigned to two methylenes (H-3 and H-21). The two doublets appeared at δ_H_3.79 and δ_H_3.91, respectively, belong to two hydrogen atoms of the methylene (H-12) which is between the two benzene rings. Because of the asymmetry of 2,4-MDIBO structure, the space environment of the two hydrogen atoms in the methylene (H-12) is different and their chemical shifts are different either. The two  triplet peaks in the range of δ_H_4.0～4.08 belong to two groups of methylenes (H-4 and H-20) which are connected with oxygen. The peaks in the range of δ_H_7.03～7.48 belong to eight hydrogen atoms on two benzene rings. Two single peaks at δ_H_8.83 and δ_H_9.50 belong to the hydrogen atoms (H-24 and H-25) attached to nitrogen atom. All NMR analytical data of 2,4-MDI_BO are listed in the Table 4 in the text

**3) ^13^C and ^1^H NMR spectra of HDI_BO**

**
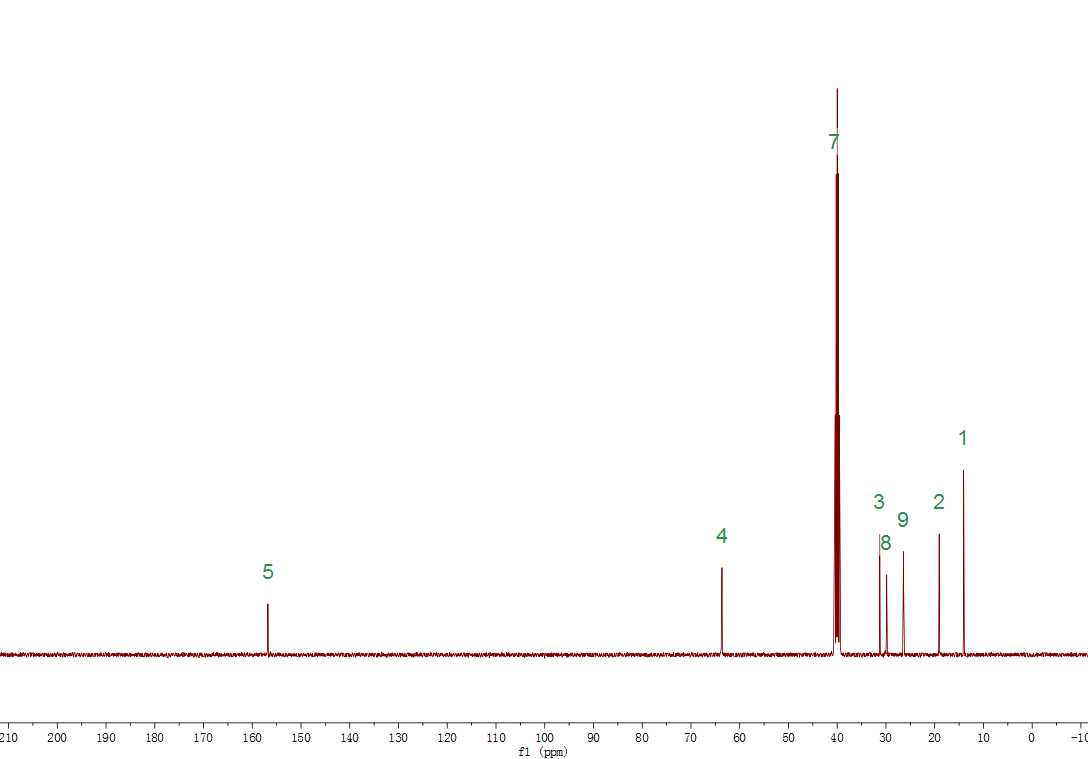
**

**Figure S9. ^13^C NMR spectrum of HDI_BO**

**
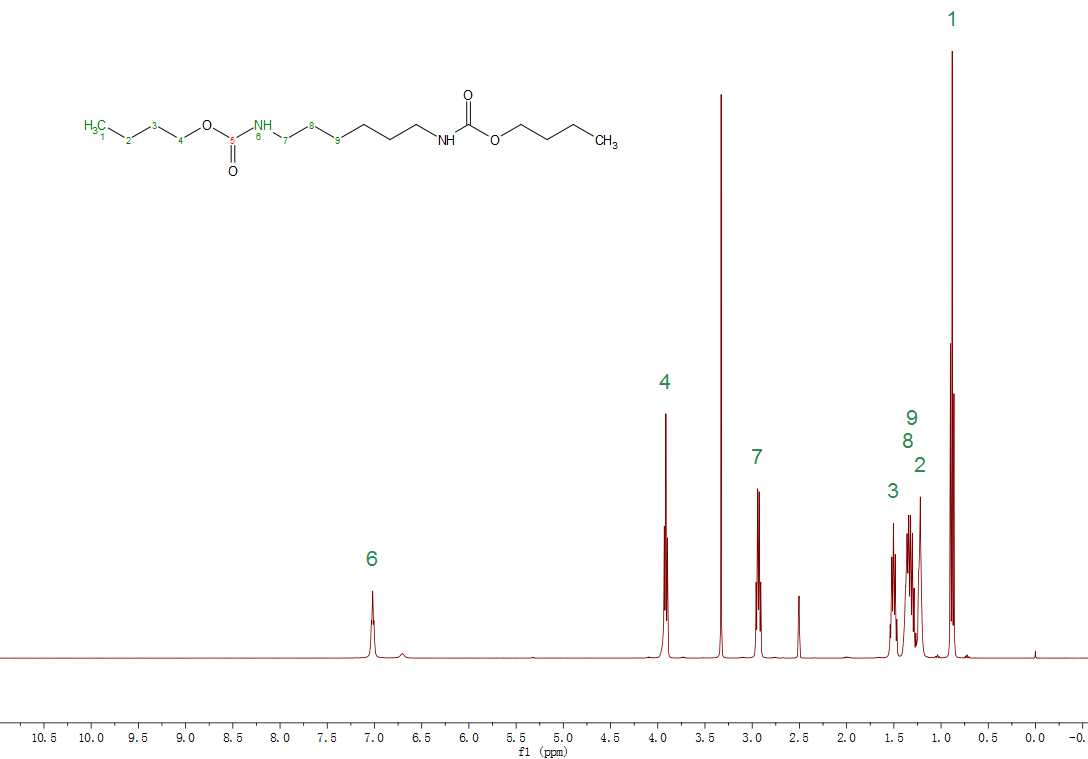
**

**Figure S10. ^1^H NMR spectrum of HDI_BO**

HDI-BO is symmetrical structure (Figure 1), there are 8 groups of hydrogen peaks and 10 carbon peaks in ^1^H and ^13^C NMR spectra, which can be easily distinguished.

In the ^13^C NMR spectrum (Figure S9), the peak at δ_C_14.04 was attributed to the methyl carbon (C-1). The peak at δ_C_19.07 was assigned to the methylene carbon(C-2). The peak at δ_C_31.27 was assigned to the methylene carbon(C-3). The peak at δ_C_63.64 belongs to the methylene carbon (C-4) which is connected with oxygen. The peak at δ_C_156.80 belongs to the carbonyl carbon(C-5). The peak at δ_C_39.99 belongs to the methylene carbon (C-7) and is hidden in the solvent peak of DMSO, whose chemical shift is at the relatively lower field because it is adjacent to nitrogen atom. The peaks at δ_C_29.85 and δ_C_26.39 belong to two methylene carbons (C-8 and C-9), respectively.

In the ^1^H NMR spectrum (Figure S10), the triplet at δ_H_0.88 was attributed to methyl (H-1). The multiple peaks in the range of δ_H_1.29～1.36 were assigned to two methylenes (H-2 and H-8) whose chemical shifts are too close to overlap together. The multiple peaks at δ_H_1.51 were assigned to the methylene (H-3). The triplet peak at δ_H_3.92 belongs to the methylene (H-4) which connect to oxygen. The triplet at δ_H_7.02 belongs to the hydrogen attached to nitrogen atom, which is divided into the triplet peaks because of the coupling of two hydrogen atoms on the methylene (H-7) linked to the nitrogen atom. The multiple peaks at δ_H_2.94 belong to the methylene (H-7), whose chemical shift is at the relatively low field because of the electron withdrawing effect of the adjacent nitrogen atom. The multiple peaks at δ_H_1.24 belongs to the methylene (H-9). All NMR analytical data of HDI-BO are listed in the Table 5.

**4) ^13^C and ^1^H NMR spectra of HMDI_BO**

**
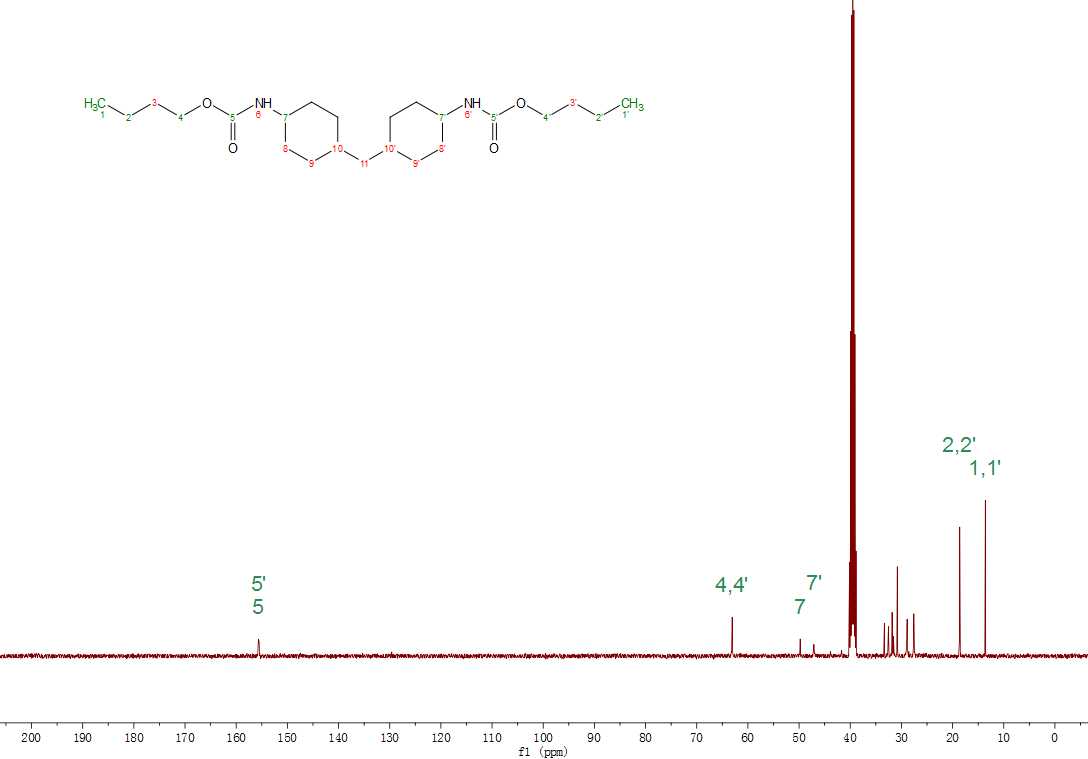
**

**Figure S11. ^13^C NMR spectrum of HMDI_BO**

**
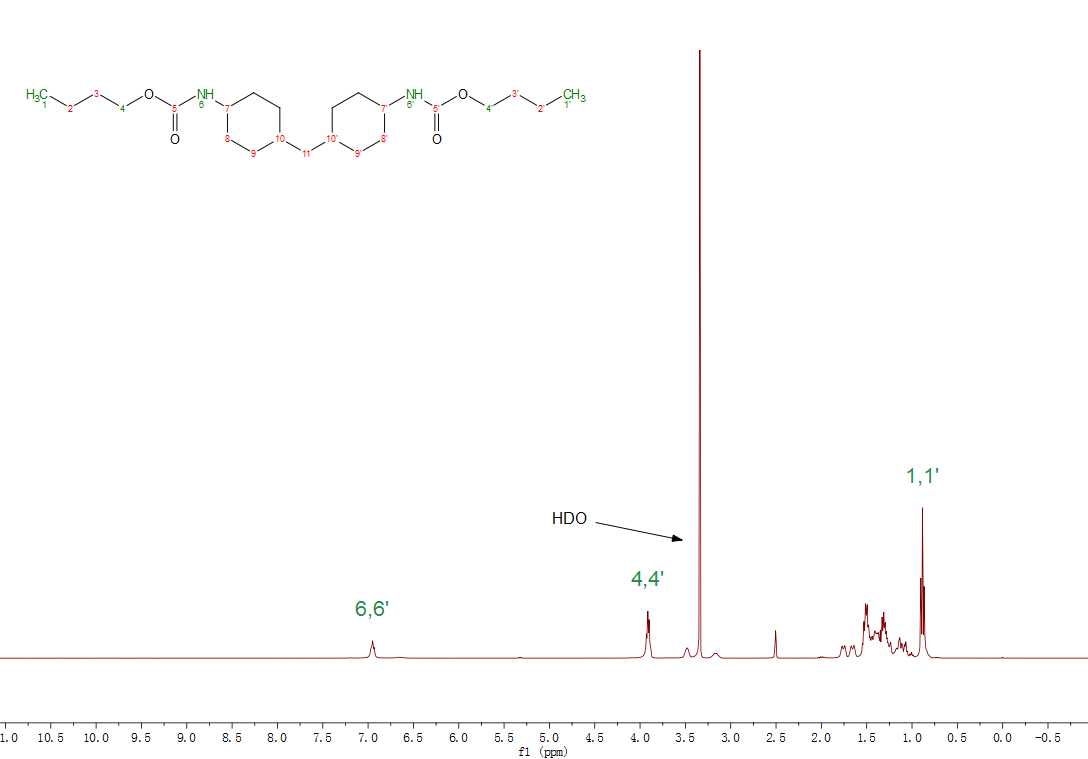
**

**Figure S12. ^1^H NMR spectrum of HMDI_BO**

In the HMDI-BO structure (Figure 1), there are two six-member rings of the cyclohexane. Because the six-member rings of the cyclohexane is not a planar ring, thus there exist two possible conformations, in which the seemingly symmetrical atoms is asymmetry in the spatial structure, which can also be verified from its ^1^H and ^13^C NMR spectra.

In the ^13^C NMR spectrum (Figure S11), the peak at δ_C_14.02 was attributed to two methyl carbons (C-1 and C-1’). The peak at δ_C_19.09 was assigned to two methylene carbons (C-2 and C-2’). The peaks at δ_C_28.00, δ_C_28.07, δ_C_29.37, δ_C_31.29, δ_C_32.04, δ_C_32.26, δ_C_32.31, δ_C_33.02 and δ_C_33.81 were assigned to (C-3, C-3’ and C-11) and the carbons on two cyclohexanes (C-8, C-8’, C-9, C-9’, C-10 and C-10’), altogether nine carbons. The peaks at δ_C_57.27 andδ_C_47.57 belong to two carbons (C-7 and C7’) which are connected with nitrogen atom. The peak at δ_C_63.56 belongs to two methylene carbons (C-4 and C-4’) which are adjacent to oxygen atom. The peaks at δ_C_156.01 and δ_C_156.15 belong to two carbonyl carbons (C-5 and C-5’). The chemical shift difference between C-5 and C-5’ or between C-7 and C7’ also proves that the spatial structure of HMDI-BO is asymmetry.

In the ^1^H NMR spectrum (Figure S12), the multiple peaks in the range of δ_H_0.88～0.90 were attributed to two methyl groups (H-1 and H-1’). The absorption peaks overlap each other and is difficult to be identified one by one in the range of δ_H_1.05～1.75, which were assigned to several groups of methylene hydrogen atoms (H-2, H-2’, H-3, H-3’ and H-11) and the hydrogen atoms on two cyclohexane rings (H-8, H-8’, H-9, H-9’, H-10 and H-10’). The multiple peaks at δ_H_6.93 were assigned to the hydrogen atoms attached to nitrogen atom (H-6 and H-6’). In H-HCOSY spectrum (Figure S13), the peak at δ_H_6.93 is related to δ_H_3.17 and δ_H_3.48, respectively, so the peaks at δ_H_3.17 and δ_H_3.48 belong to two groups of methine (H-7 and H-7’) which are connected with nitrogen atom. This result proves that the spatial structure of HMDI-BO is asymmetry. The peaks in the range of δ_H_3.89～3.93 belong to two methylene groups (H-4 and H-4’) which are attached to oxygen atom. All NMR analytical data of HMDI-BO are listed in Table 6 in the text.

**3. X-ray figures**


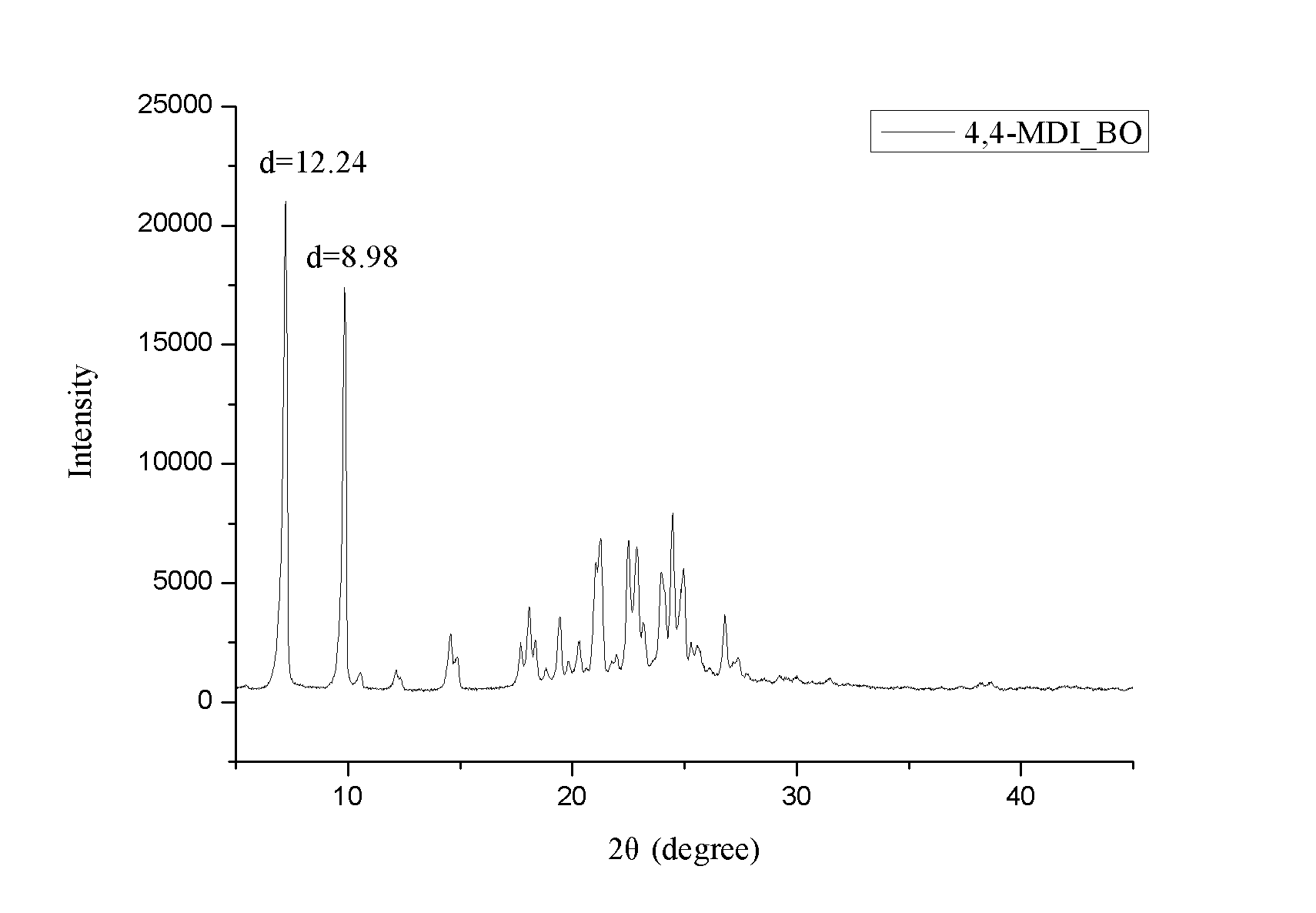


**Figure S13. X-ray spectrum of 4,4-MDI_BO**


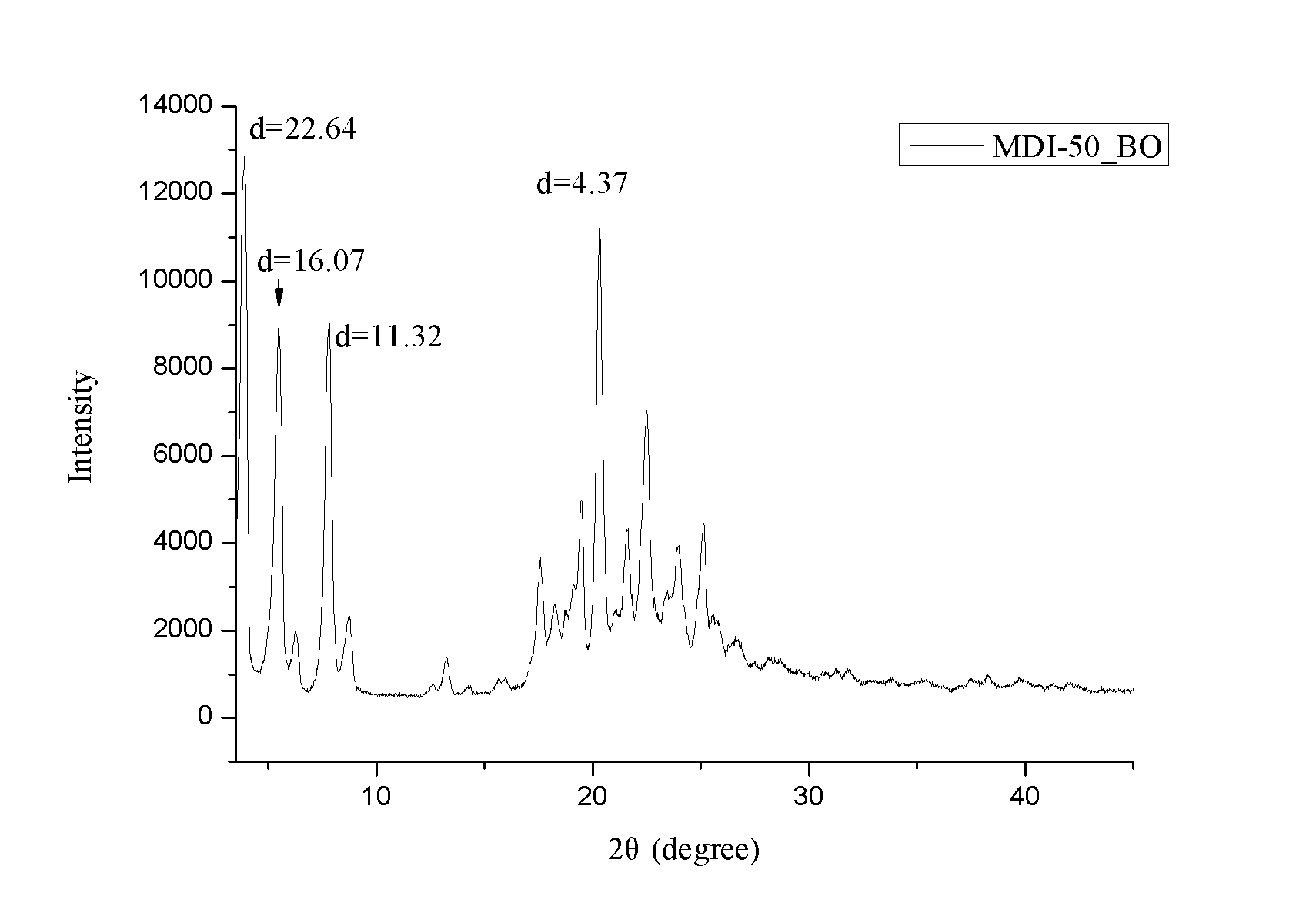


**Figure S14. X-ray spectrum of MDI-50_BO**


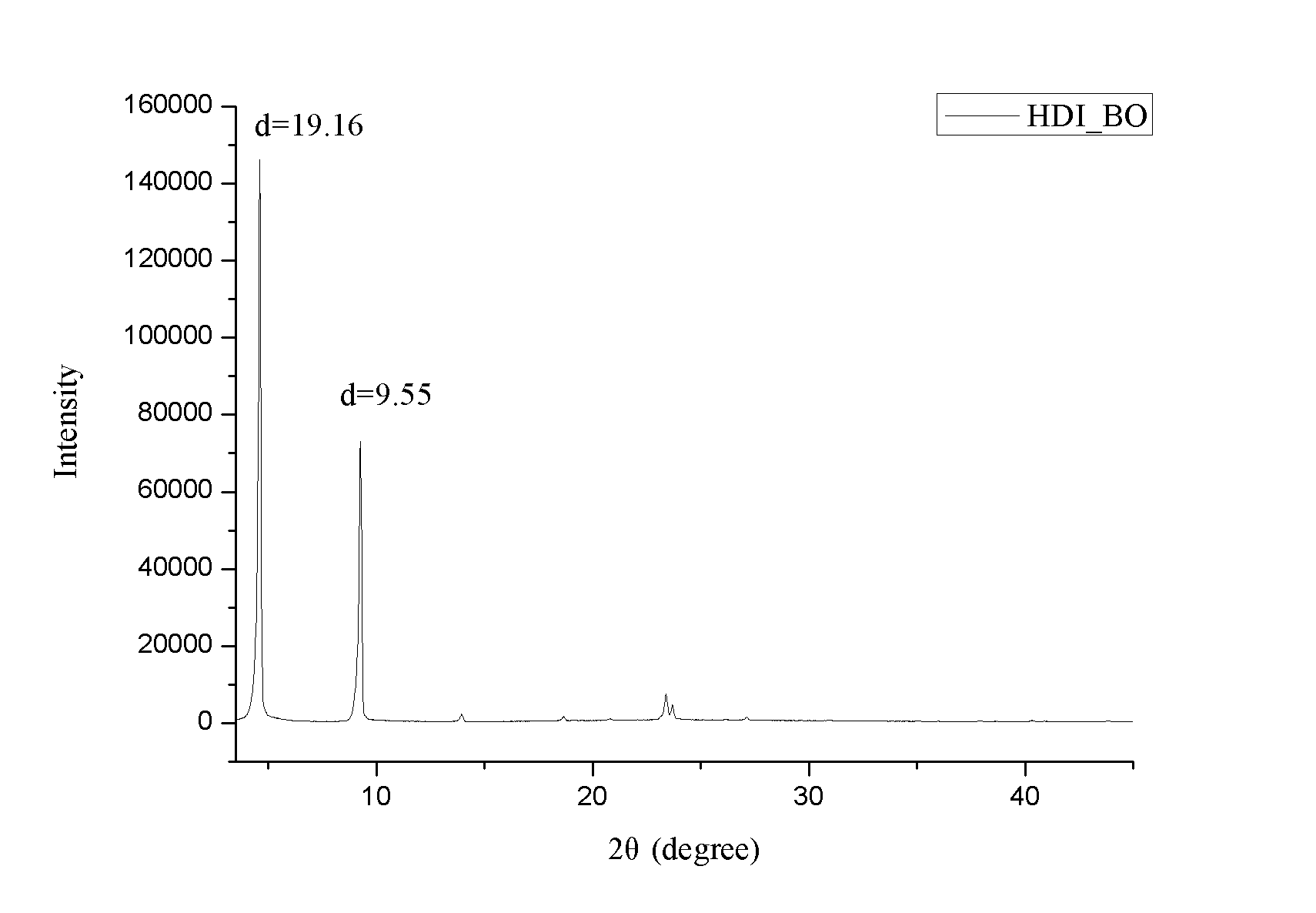


**Figure S15. X-ray spectrum of HDI_BO**


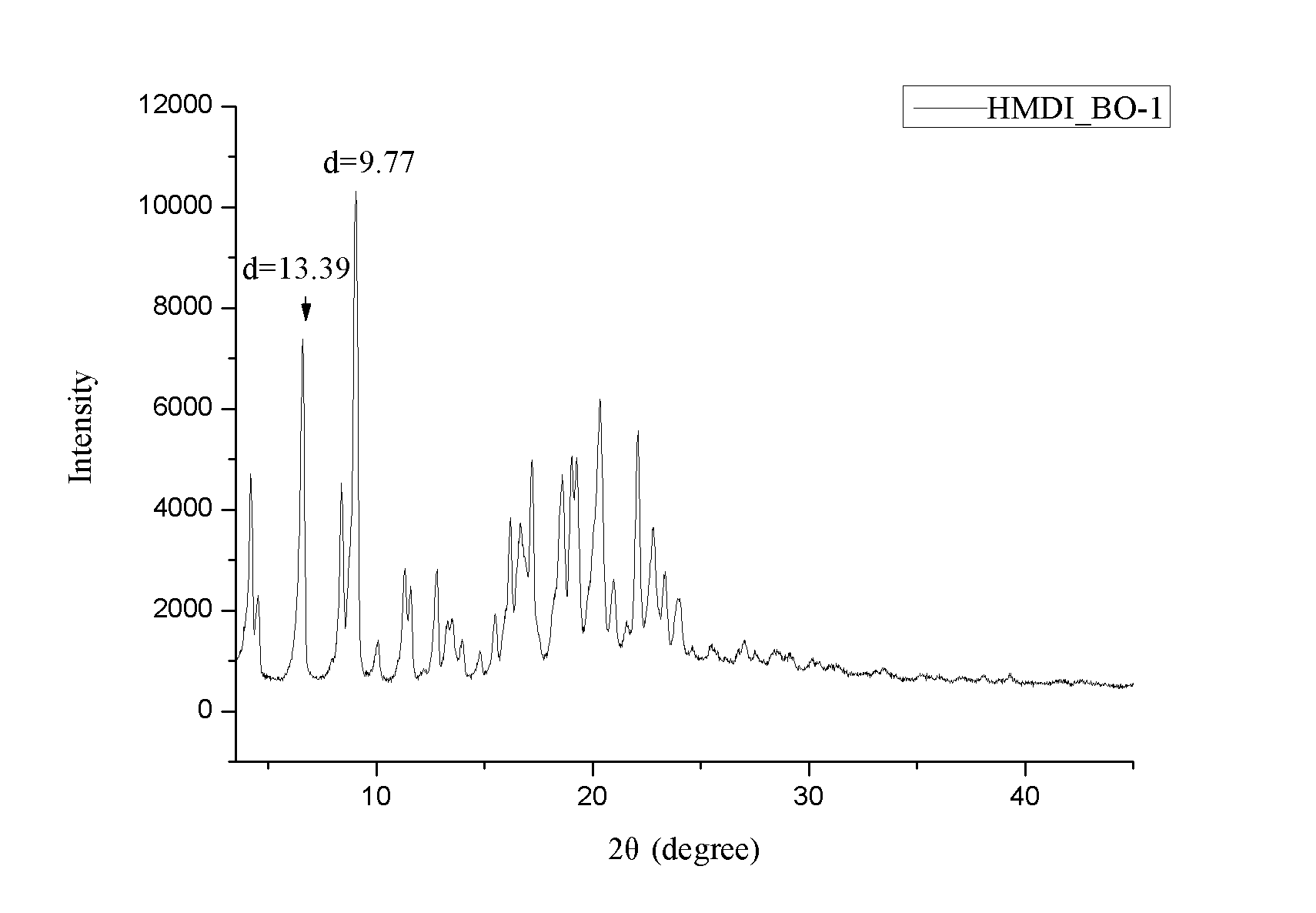


**Figure S16. X-ray spectrum of HMDI_BO-1**
