# Supplementary figures and images for: Synthesis and structure/properties characterizations of four polyurethane model hard segments
Source: R Soc Open Sci. 2018 Jul 25;5(7):180536. doi: 10.1098/rsos.180536 (PMC6083698; doi:10.1098/rsos.180536)

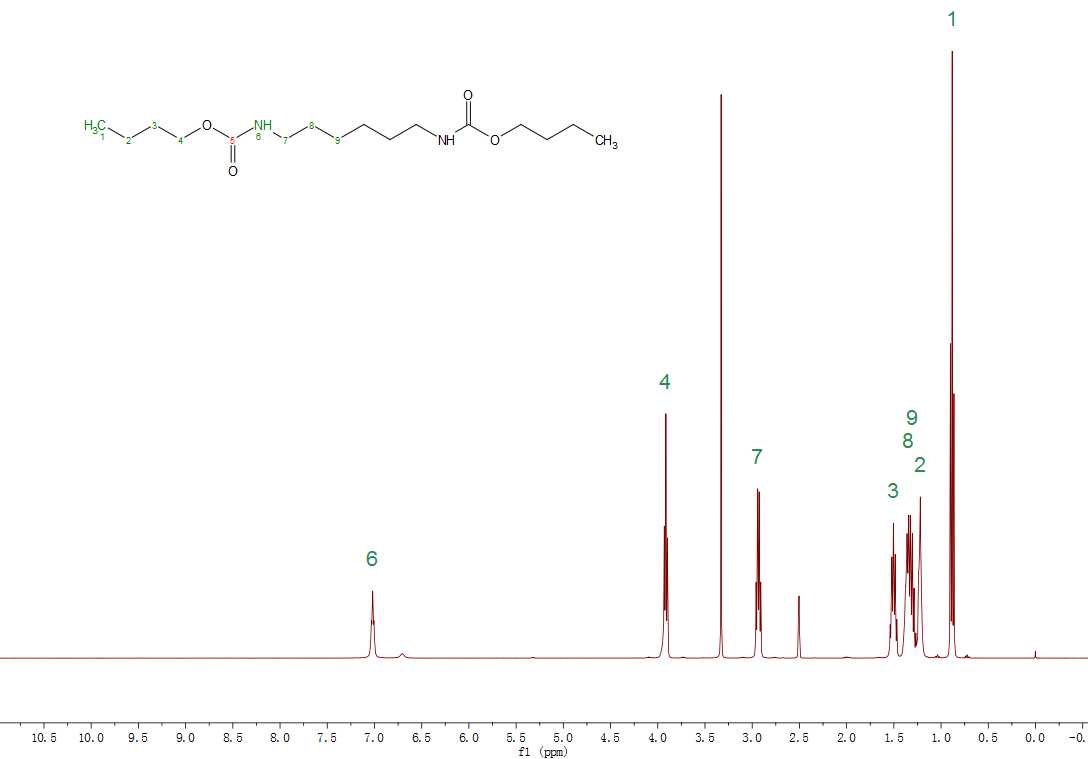

Supplement: Figure S1.tif [file rsos180536supp2.jpeg]

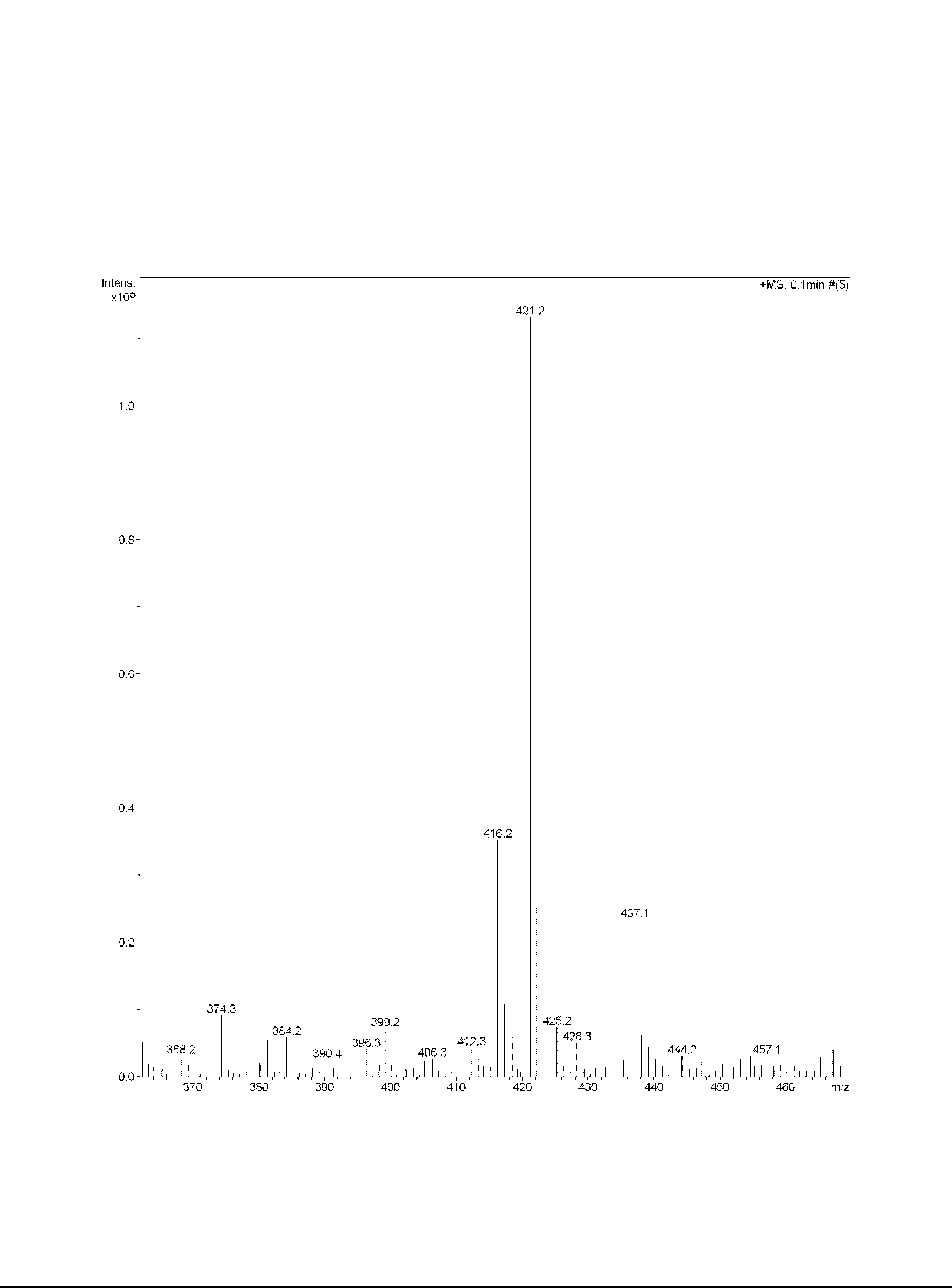

Supplement: Figure S2.tif [file rsos180536supp3.tif]

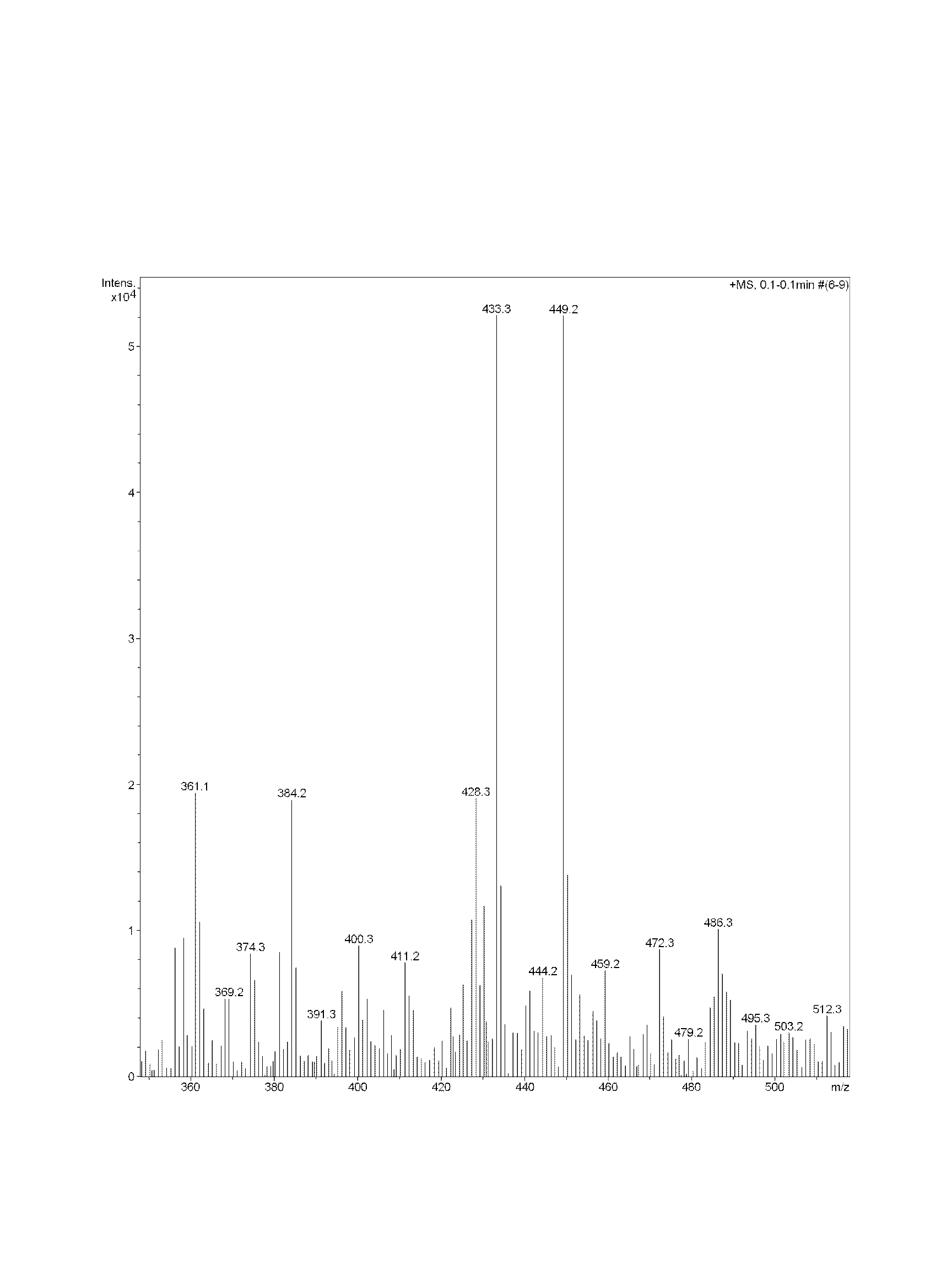

Supplement: Figure S3.tif [file rsos180536supp4.tif]

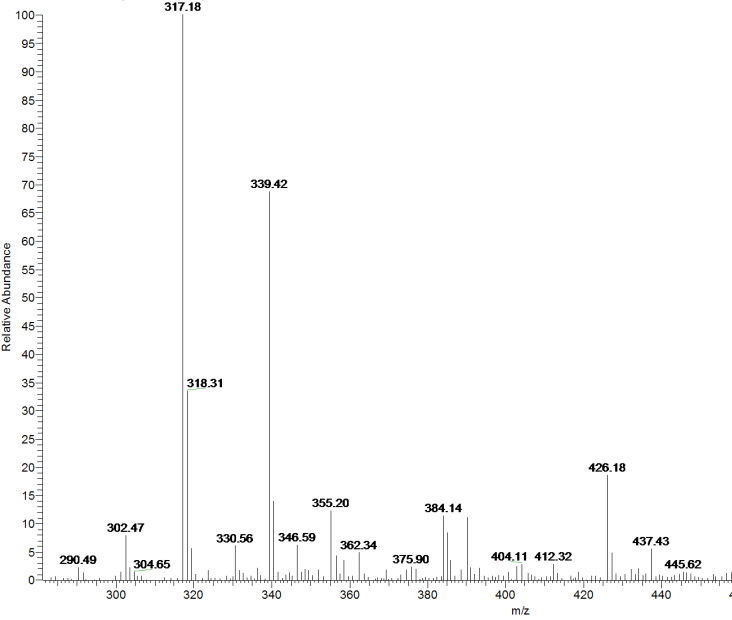

Supplement: Figure S4.tif [file rsos180536supp5.tif]

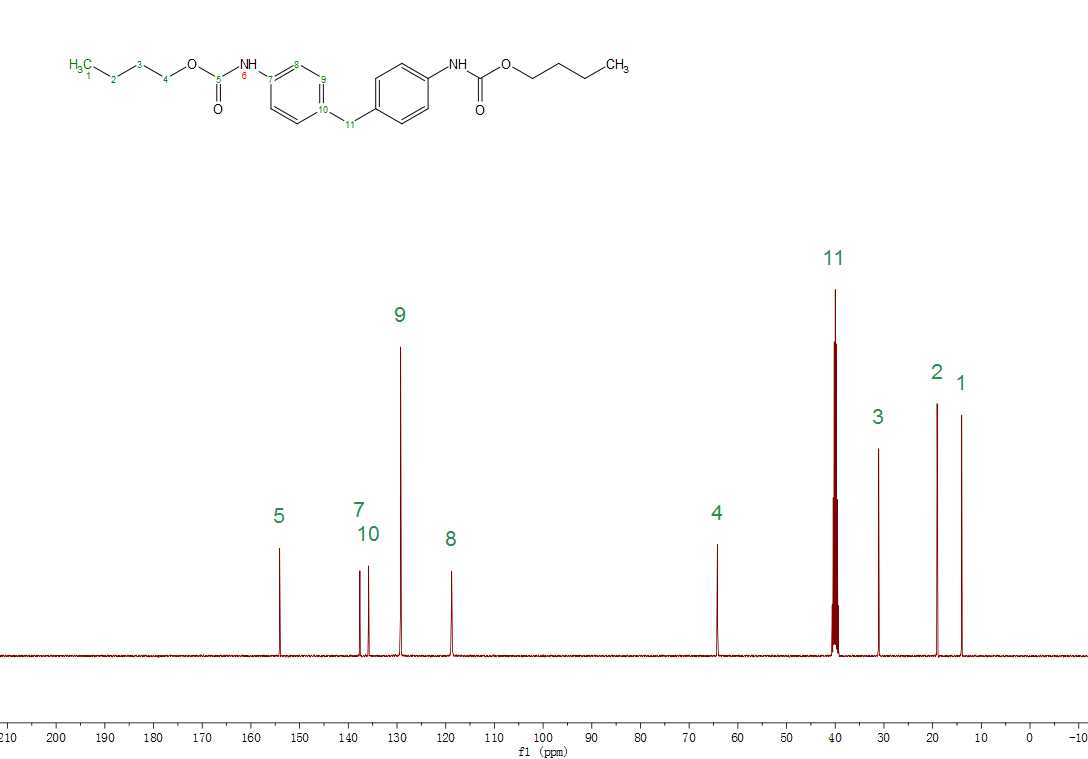

Supplement: Figure S5.jpeg [file rsos180536supp6.jpeg]

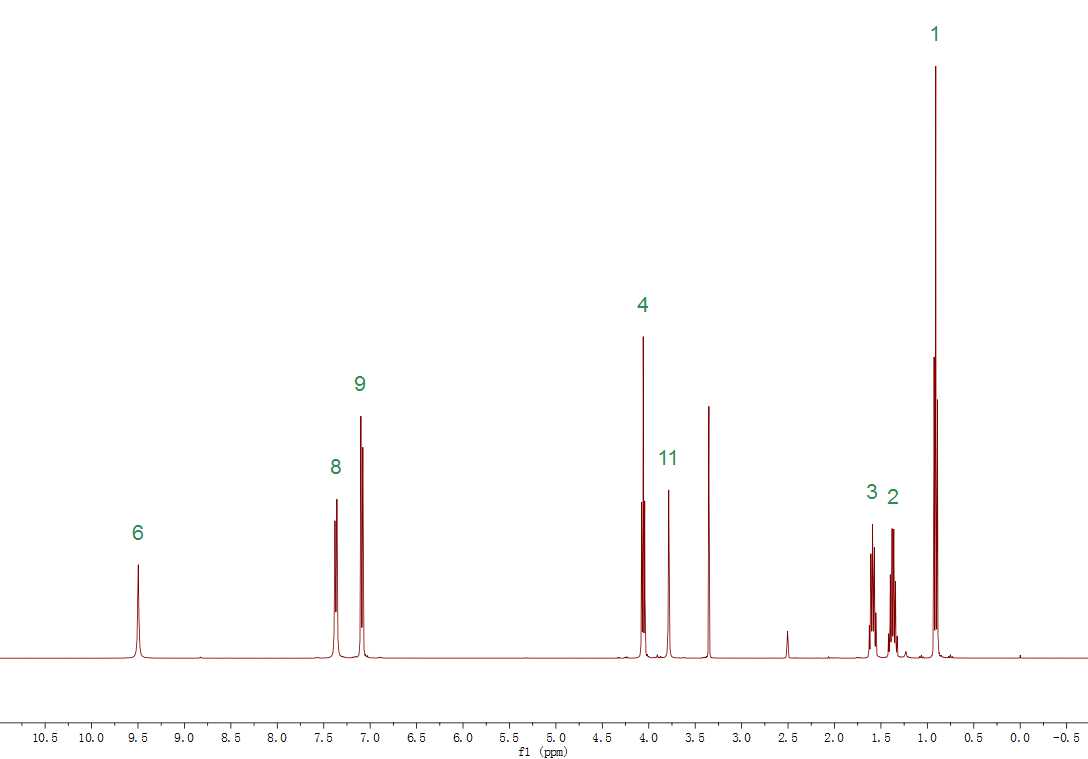

Supplement: Figure S6.jpeg [file rsos180536supp7.jpeg]

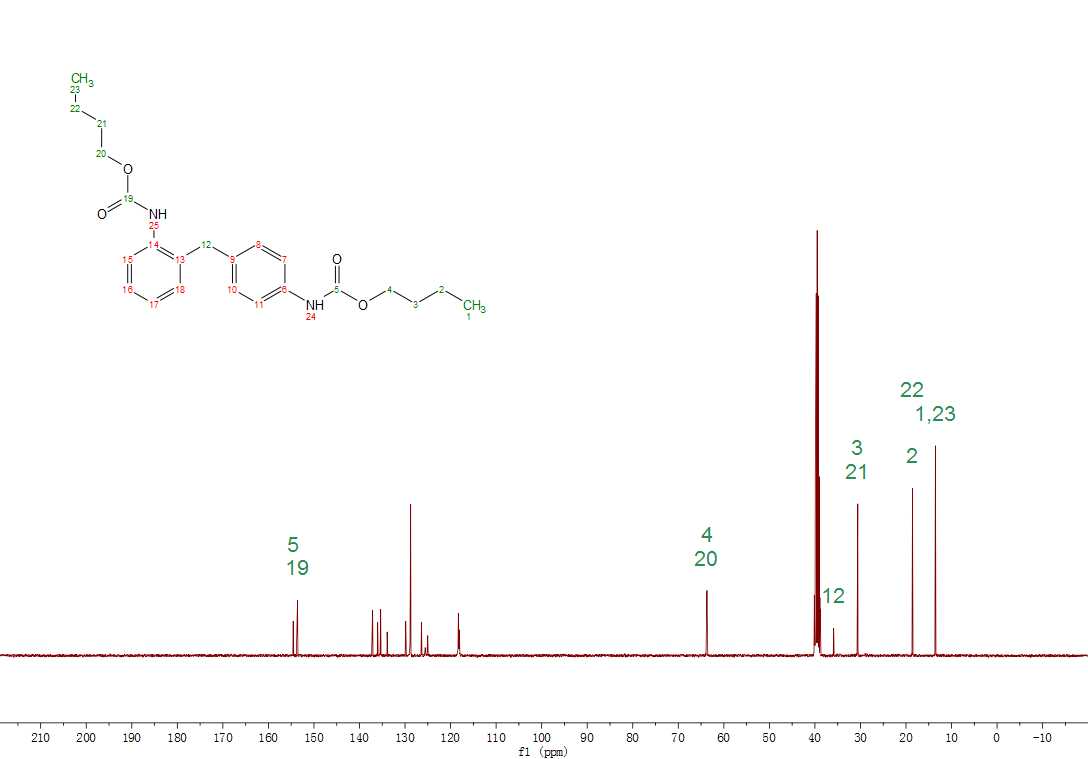

Supplement: Figure S7.jpeg [file rsos180536supp8.jpeg]

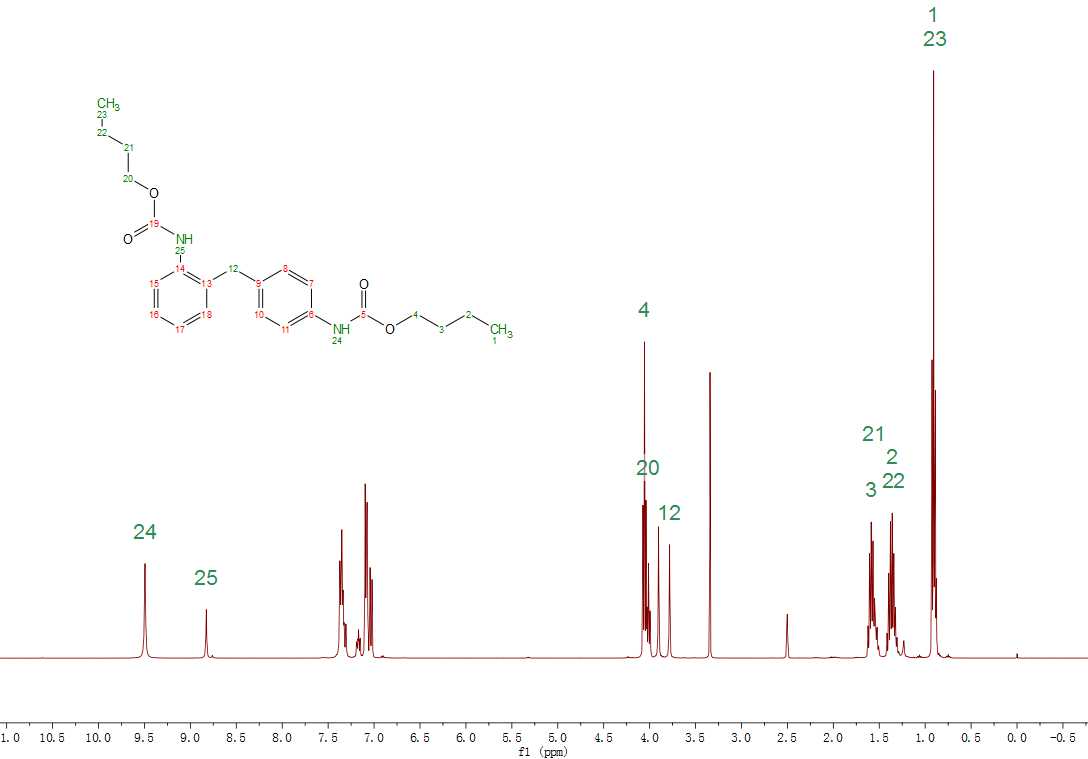

Supplement: Figure S7.jpeg [file rsos180536supp9.jpeg]

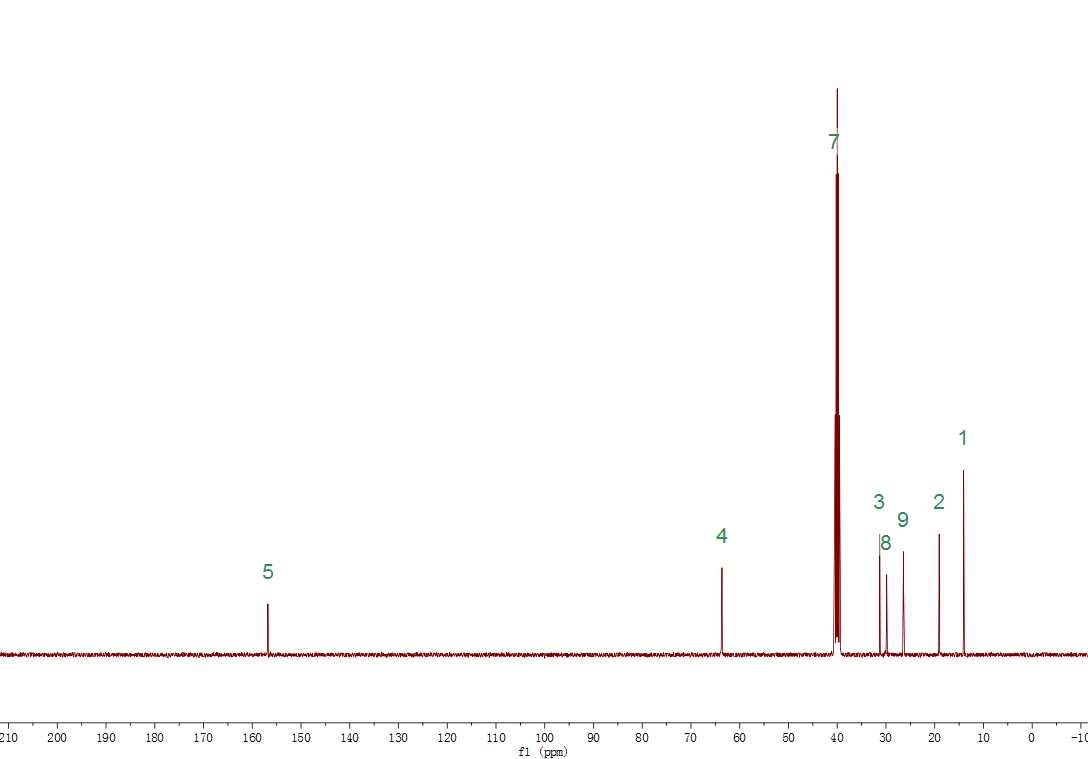

Supplement: Figure S8.jpeg [file rsos180536supp10.jpeg]

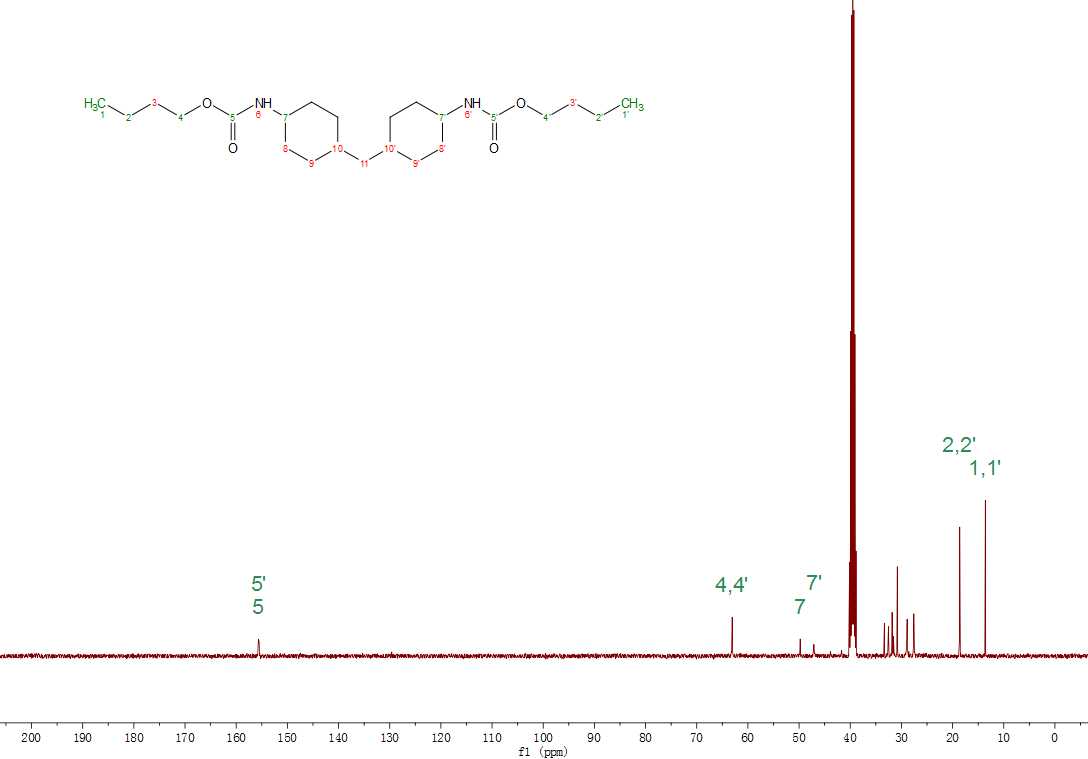

Supplement: Figure S10.jpeg [file rsos180536supp12.jpeg]

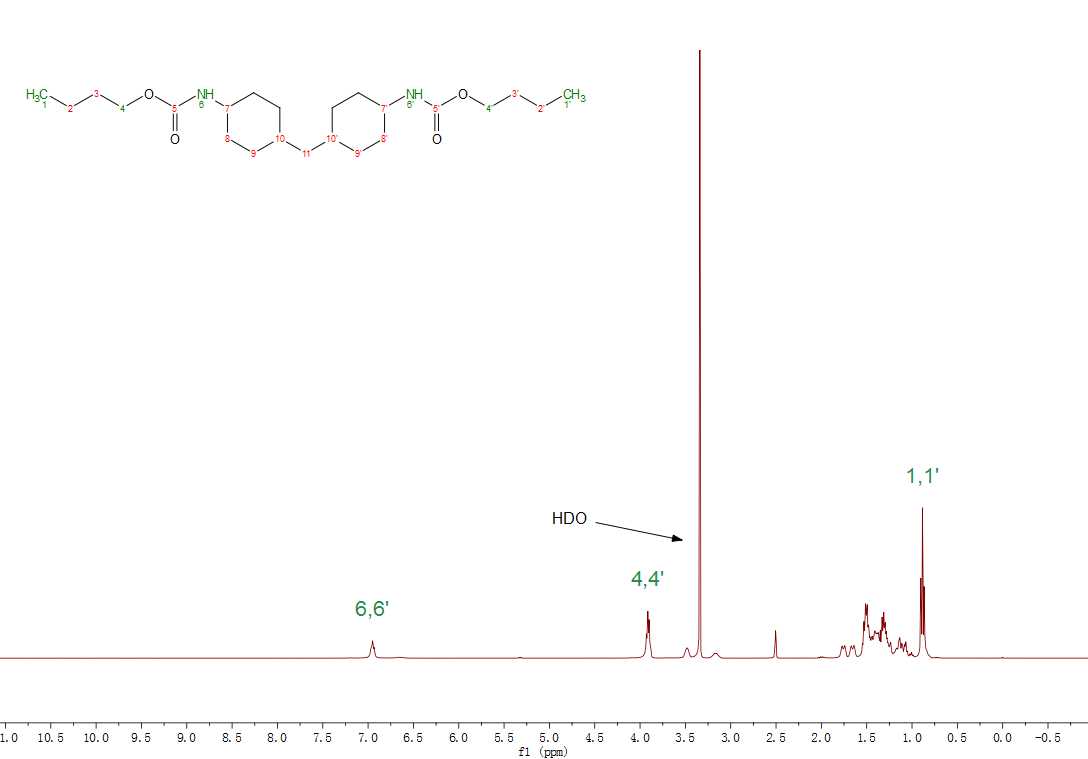

Supplement: Figure S11.jpeg [file rsos180536supp13.jpeg]

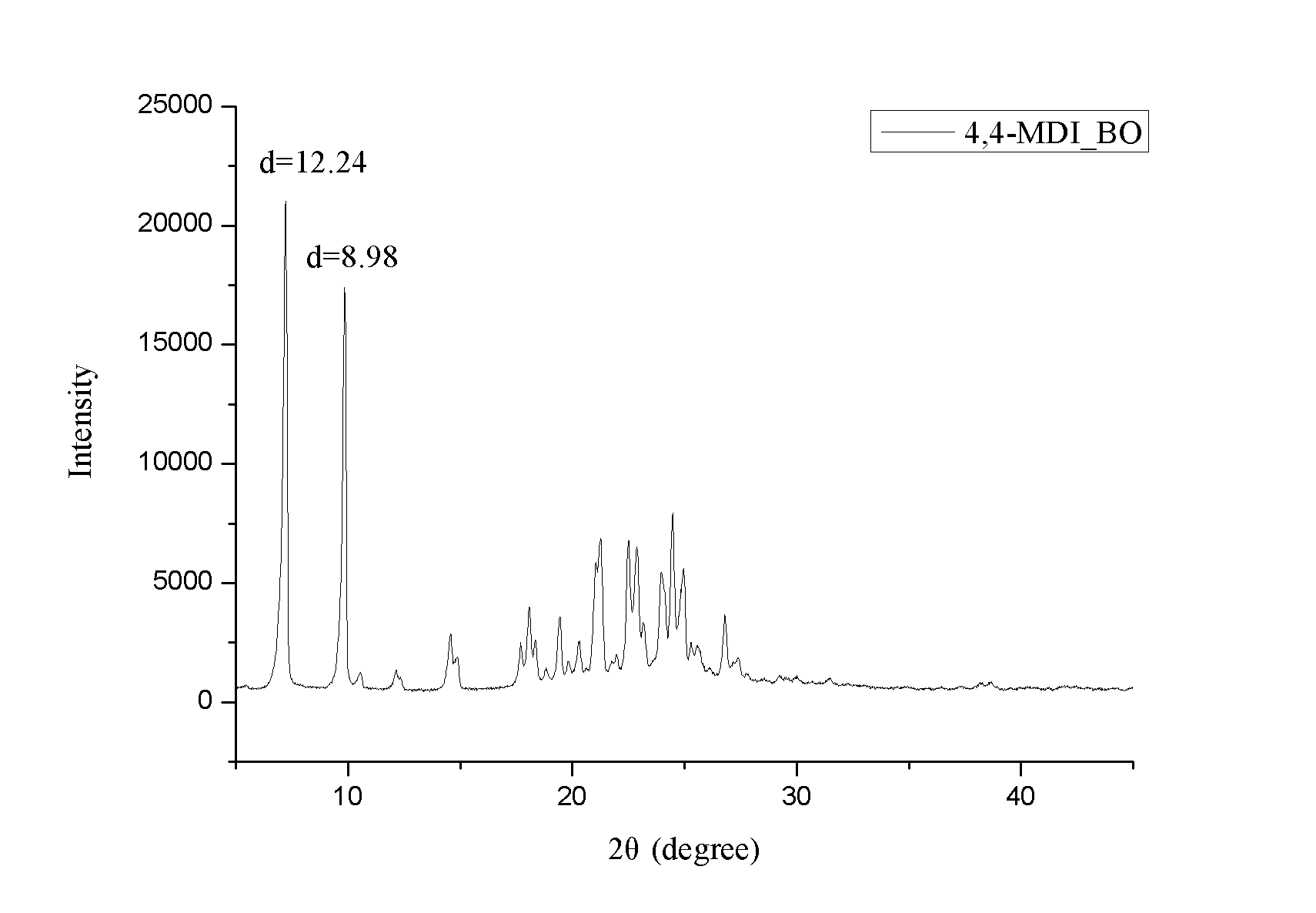

Supplement: Figure S13.gif [file rsos180536supp14.gif]

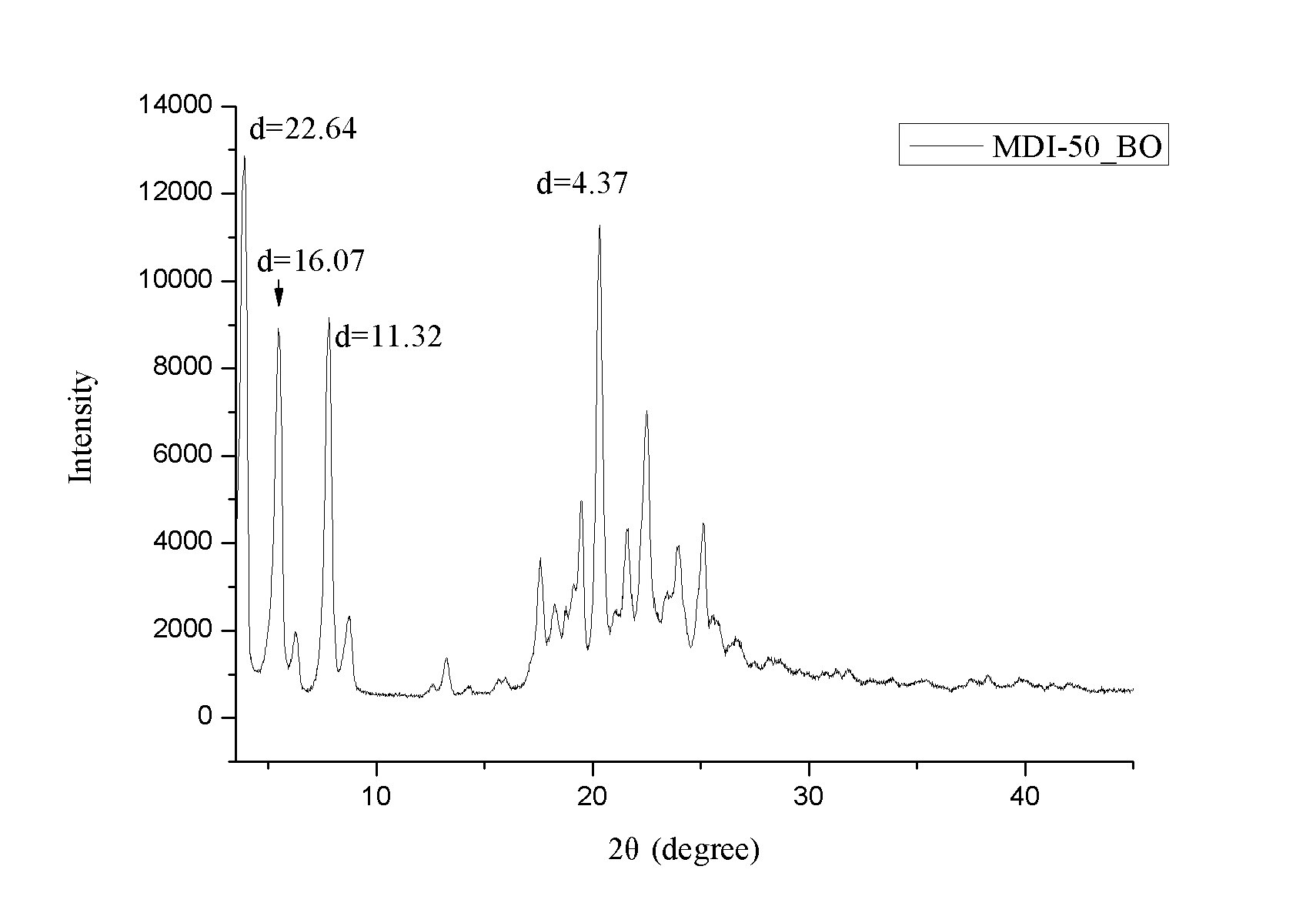

Supplement: Figure S14.gif [file rsos180536supp15.gif]

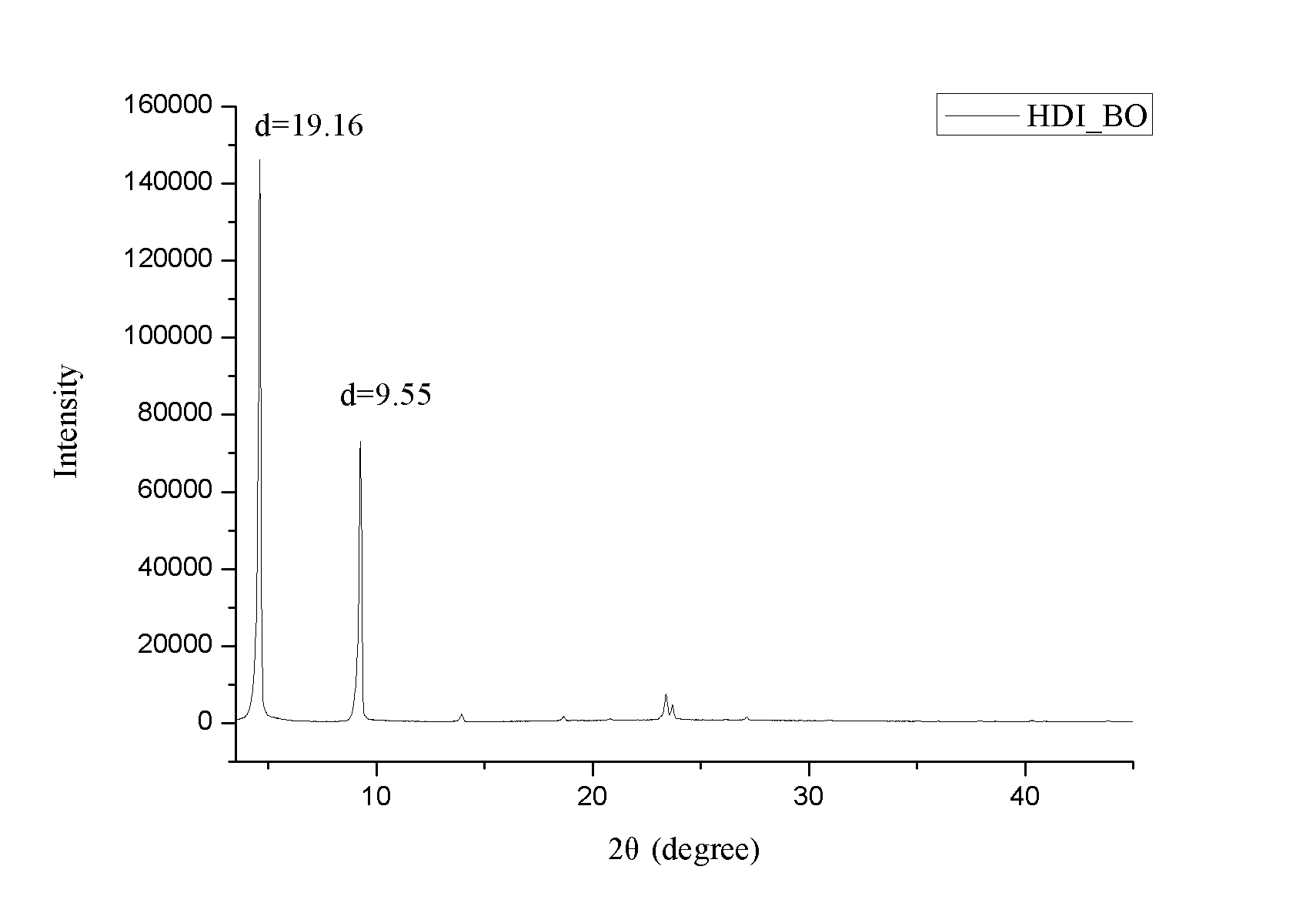

Supplement: Figure S15.gif [file rsos180536supp16.gif]

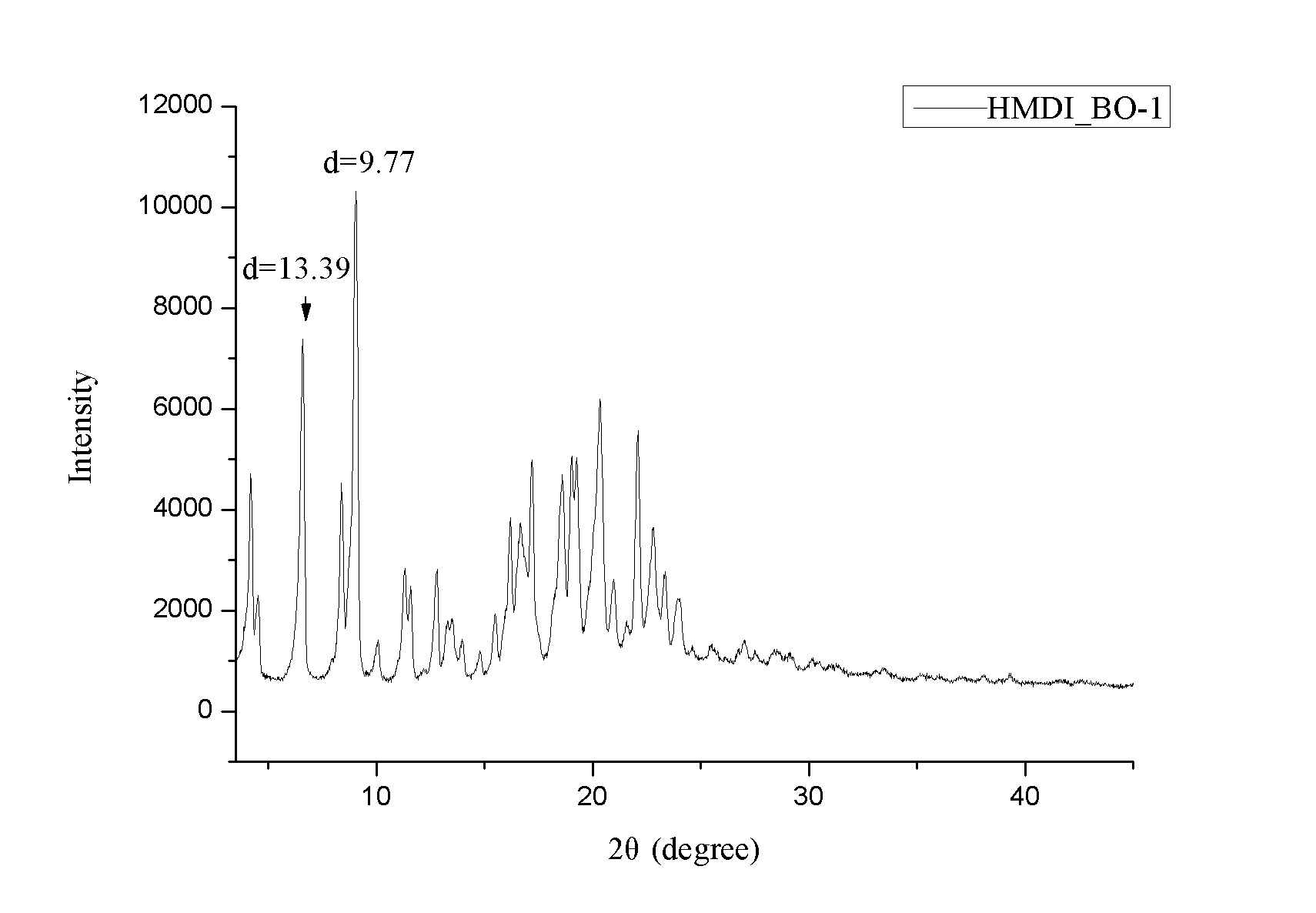

Supplement: Figure S16.gif [file rsos180536supp17.gif]
